# Macrotroponin Complexes and Risk of Cardiovascular Events

**DOI:** 10.1161/CIRCULATIONAHA.126.080483

**Published:** 2026-09-08

**Authors:** Marie de Bakker, Alexander J.F. Thurston, Ola Hammarsten, Mareta Khachatryan, Takeshi Fujisawa, Ziwen Li, John W. McEvoy, Bertil Lindahl, Torbjørn Omland, Naveed Sattar, Cathie L.M. Sudlow, Kai M. Eggers, Paul Welsh, Nicholas L. Mills

**Affiliations:** 1 British Heart Foundation Centre of Research Excellence, Institute for Neurosciences and Cardiovascular Research (M.d.B., A.J.F.T., T.F., Z.L., N.L.M.), University of Edinburgh 523476 , Edinburgh, United Kingdom.; 2 Usher Institute (M.d.B., C.L.M.S.), University of Edinburgh 523476 , Edinburgh, United Kingdom.; 3 British Heart Foundation Cardiovascular Biomarker Laboratory (T.F.), University of Edinburgh 523476 , Edinburgh, United Kingdom.; 4 Department of Clinical Chemistry, Sahlgrenska University Hospital, Göteborg, Sweden (O.H., M.K.).; 5 National Institute for Prevention and Cardiovascular Health and University of Galway School of Medicine, Galway, Ireland (J.W.M.).; 6 Division of Cardiology, Johns Hopkins School of Medicine, Johns Hopkins University, Baltimore, MD (J.W.M.).; 7 Department of Epidemiology and the Welch Center for Prevention, Epidemiology, and Clinical Research, Johns Hopkins Bloomberg School of Public Health, Baltimore, MD (J.W.M.).; 8 Department of Medical Sciences and Uppsala Clinical Research Center, Uppsala University, Uppsala, Sweden (B.L., K.M.E.).; 9 Department of Cardiology, Akershus University Hospital, Lørenskog, Norway (T.O.).; 10 K.G. Jebsen Center for Cardiac Biomarkers, University of Oslo, Oslo, Norway (T.O.).; 11 School of Cardiovascular and Metabolic Health, University of Glasgow, Glasgow, United Kingdom (N.S., P.W.).

**Keywords:** biomarkers, cardiovascular diseases, risk, troponin

## Abstract

**BACKGROUND:**

Cardiac troponin is increasingly used for cardiovascular risk prediction. While abnormal values often reflect subclinical cardiovascular disease, they can be raised due to interference, where antibodies influence the assay or clearance of troponin from the circulation by forming macrotroponin complexes. The frequency of macrotroponins and its association with future risk are unknown.

**METHODS:**

In a general population cohort, participants with both cardiac troponin I and T measurements available (n=19 499, median 49 [25th–75th percentile, 36–58] years of age, 58.3% women) were categorized into 3 groups: no myocardial injury (neither cardiac troponin I or T was elevated), suspected macrotroponin (either cardiac troponin was elevated and discordant with a >3-fold difference in concentration), and myocardial injury (either cardiac troponin was elevated without discordance). Macrotroponin complex formation was verified in a subset of samples (n=203) using immunoglobulin depletion and ultracentrifugation. Associations with future myocardial infarction, ischemic stroke, or cardiovascular death were evaluated using Cox proportional hazards models adjusted for known cardiovascular risk factors. The generalizability of our findings was determined through replication in a second general population cohort study.

**RESULTS:**

Elevated cardiac troponin I was present in 1% (193 of 19 499) of participants and elevated cardiac troponin T in 6% (1159 of 19 499). Among those, 48% and 57% were discordant, respectively. Biochemical testing attributed 92.5% of discordant troponin I and 15.4% of discordant troponin T to macrotroponin complexes. Participants with myocardial injury were at increased risk of future cardiovascular events compared with those without (cardiac troponin I: adjusted hazard ratio [HR], 2.90 [95% CI, 1.99–4.22]; cardiac troponin T: adjusted HR, 1.99 [95% CI, 1.63–2.42]), whereas those with suspected macrotroponin had similar risk as those without elevated values for suspected macrotroponin I (adjusted HR, 1.59 [95% CI, 0.94–2.70]) and T (adjusted HR, 1.27 [95% CI, 1.00–1.61]). Findings were similar in the replication cohort.

**CONCLUSIONS:**

Assay interference due to macrotroponin complex formation is common in individuals with elevated cardiac troponin concentrations in the general population, particularly for cardiac troponin I. This has important implications for the use of cardiac troponin testing for cardiovascular risk assessment in the general population.

**Clinical Perspective**

**What Is New?**

- We report the frequency of macrotroponin complex formation and its association with risk of future cardiovascular events in the general population.
- Assay interference due to macrotroponin complex formation is common; approximately half of the individuals with elevated cardiac troponin I or T concentrations have discordance between assays, with biochemical analysis confirming macrotroponin in 93% of discordant troponin I samples and 15% of discordant troponin T samples.
- Participants with elevated cardiac troponin concentrations without discordance were at increased risk of future cardiovascular events, whereas those with assay discordance (and interference was therefore suspected) had similar risk as those without elevated troponin values.

**What Are the Clinical Implications?**

- Assay interference due to macrotroponin complex formation may account for a substantial proportion of elevated cardiac troponin results in asymptomatic individuals from the general population.
- Failure to recognize assay interference and macrotroponins may lead to unnecessary cardiac investigations and prescribing when cardiac troponin is used for risk assessment.
- We would recommend that, in asymptomatic individuals with elevated cardiac troponin concentrations, testing be repeated using an alternative assay or that samples be referred for macrotroponin analysis at a specialized laboratory before considering cardiac imaging for the presence of subclinical cardiovascular disease.

Cardiac troponins are sarcomeric proteins released into the circulation following injury, and their quantification using immunoassays is integral to the clinical diagnosis of myocardial infarction. The development of high-sensitivity assays with enhanced analytical precision at low concentrations has led to new clinical indications for cardiac troponin testing to support cardiovascular risk prediction in the general population.^1–12^ However, adoption has been limited in part by concerns about the clinical implications of identifying asymptomatic individuals with abnormal values. While abnormal values may reflect subclinical cardiovascular disease, cardiac troponin can be raised due to interferences where antibodies influence the assay or clearance of troponin from the circulation by forming macrotroponin complexes (Figure 1).^13–15^ Measuring cardiac troponin with an alternative assay can help to differentiate those with myocardial injury from those where elevations are due to interference.^16,17^ A greater than 3-fold difference in absolute concentration between 2 cardiac troponin assays has been suggested as a pragmatic approach to identify potential assay interference.^16^ To date, the frequency of macrotroponin complex formation and the implications for cardiovascular risk prediction in the general population are unknown.^18^

**Figure 1.**
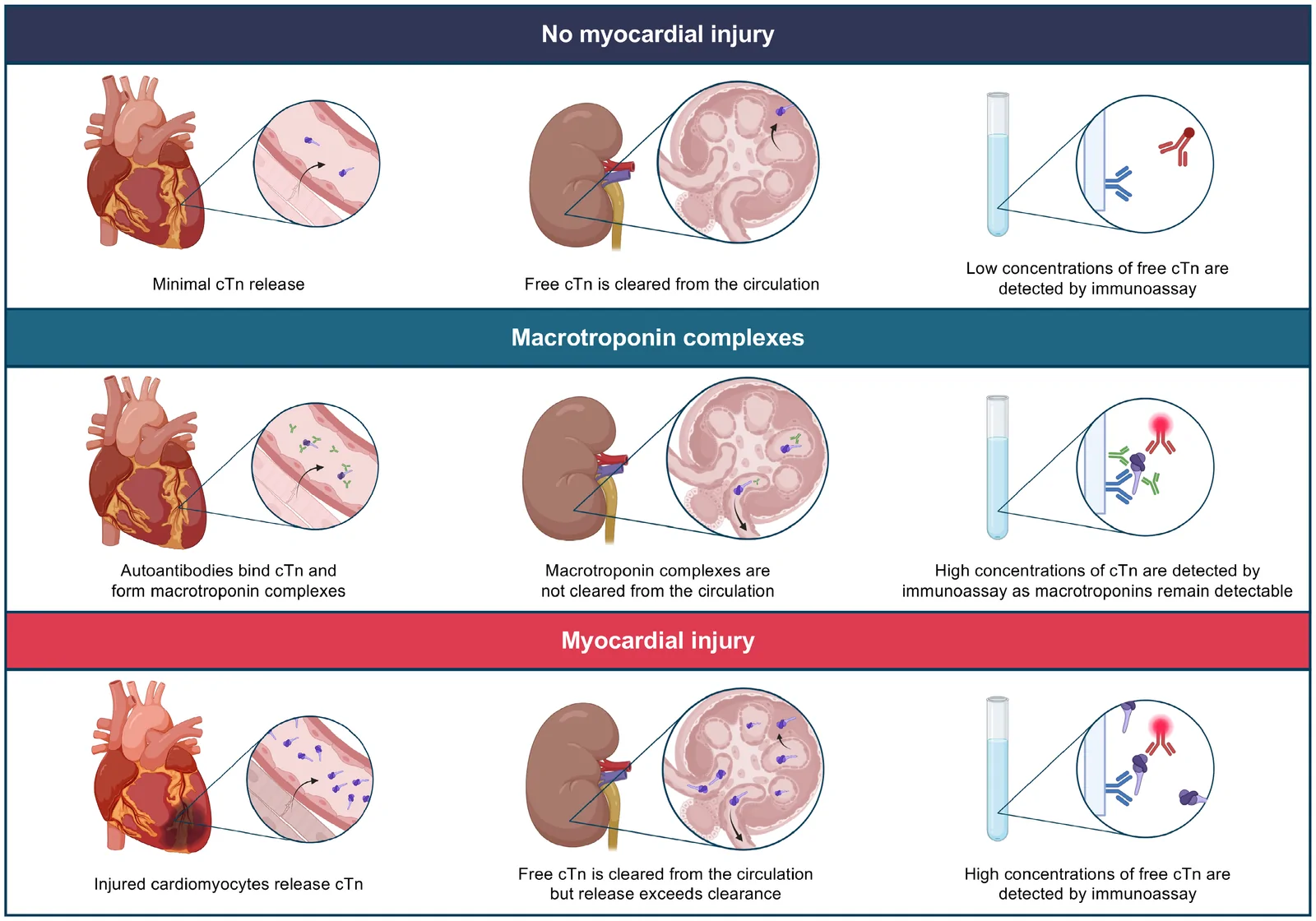
**Immunoassay interference due to formation of macrotroponin complexes.** Autoantibodies to cTn (cardiac troponin) bind to form macrotroponin complexes, which persist in the circulation for longer than free cardiac troponin, increasing the cardiac troponin concentration measured by immunoassays.

Our aim was to evaluate the frequency of macrotroponins in a general population cohort^7^ and to assess whether it is associated with risk of future cardiovascular events. Suspected macrotroponin complex formation was identified through discordance between measurements from 2 cardiac troponin assays, with verification performed in a subset of discordant samples using 2 complementary methods recommended by the International Federation of Clinical Chemistry.^16,19,20^ The generalizability of our findings was determined through replication in a second general population cohort study.

## METHODS

### Study Population

GS:SFHS (Generation Scotland:Scottish Family Health Study) is a family- and population-based cohort study that enrolled 24 090 participants (age between 18 and 98 years) via primary care general practices in Scotland between February 2006 and March 2011. More details on the selection procedure and clinical characteristics of the cohort are described elsewhere.^7^ For this study, we included individuals with available measurements of cardiac troponin I (ARCHITECT high-sensitivity troponin I assay, Abbott Diagnostics) and cardiac troponin T (Elecsys Troponin T Gen 5 STAT high-sensitivity assay, Roche Diagnostics) as well as outcome data. Study participants provided written informed consent that permitted linkage to their medical records. Ethics approval was obtained from the National Health Service Tayside Research Ethics Committee (Research Ethics Committee reference No. 05/S1401/89). The study was conducted according to principles of the Declaration of Helsinki and followed the Strengthening the Reporting of Observational Studies in Epidemiology guidelines (Supplemental Material). According to the terms of consent for Generation Scotland participants, access to data must be reviewed by the Generation Scotland Access Committee. Applications should be made to genscot@ed.ac.uk.

We replicated key aspects of our analysis in NHANES (the National Health and Nutrition Examination Survey), which is designed to be a nationally representative sample of the population in the United States. Participants were selected from a noninstitutionalized, civilian population using a complex, stratified, multistage probability cluster sampling design. More details on the selection procedure and clinical characteristics of the cohort are described elsewhere.^21^ For this study, we included individuals from NHANES 1999 to 2004 with available measurements of cardiac troponin I (ARCHITECT high-sensitivity troponin I assay, Abbott Diagnostics) and cardiac troponin T (Elecsys Troponin T Gen 5 STAT high-sensitivity assay, Roche Diagnostics) as well as outcome data. Written informed consent was obtained from all participants. The NHANES protocols and the measurement of high-sensitivity troponin in stored specimens were approved by the National Center for Health Statistics ethics review board. NHANES data, including cardiac troponin concentrations, are publicly available at the Centers for Disease Control and Prevention website (https://wwwn.cdc.gov/nchs/nhanes/).

### Cardiac Troponin Measurements

In GS:SFHS, venous blood samples were collected at baseline under fasting conditions and centrifuged, and serum was stored in aliquots at −80 °C according to a standardized protocol until batch analysis was performed between 2016 and 2017 at the University of Glasgow (Glasgow, United Kingdom). Both cardiac troponin I and T concentrations were measured at first thaw. Cardiac troponin I (ARCHITECT _*STAT*_ high-sensitivity troponin I assay, Abbott Diagnostics) and cardiac troponin T (Elecsys Troponin T high-sensitivity assay, Roche Diagnostics) were measured on i1000SR and Cobas e411 analyzers, respectively, using the manufacturers’ calibrators and quality controls. The cardiac troponin I assay has a limit of blank of 1.2 ng/L and limit of detection of 1.9 ng/L, whereas the cardiac troponin T assay has a limit of blank of 3 ng/L and limit of detection of 5 ng/L.^22^ The limit of blank is the highest apparent analyte concentration expected when multiple measurements are performed on a true blank sample (ie, a sample without the analyte). The sex-specific 99th percentile upper reference limits (URLs) are 16 ng/L and 34 ng/L in women and men, respectively, for cardiac troponin I and 9 ng/L and 16 ng/L in women and men, respectively, for cardiac troponin T.^23^ During the conduct of this study, we participated in the National External Quality Assurance Scheme for both assays.

Serum samples from participants in the NHANES study were also stored in aliquots at −80 °C according to a standardized protocol until batch analysis was performed between 2018 and 2020 at the University of Maryland School of Medicine (Baltimore, MD).^24^ Cardiac troponin I and T concentrations were measured at first thaw for the majority of the stored samples (93%). For this study, we used data from the Abbott and Roche assays that were also measured in the GS:SFHS, applying the same thresholds to allow for comparison. High-sensitivity cardiac troponin I was measured on i2000SR (ARCHITECT high-sensitivity troponin I assay, Abbott Diagnostics). A limit of blank of 1.2 ng/L and limit of detection of 1.9 ng/L were used.^25^ The sex-specific 99th percentile URLs used in the current study were 16 ng/L and 34 ng/L in women and men, respectively. High-sensitivity cardiac troponin T was measured on a Cobas e601 analyzer (Elecsys Troponin T Gen 5 STAT high-sensitivity assay, Roche Diagnostics). This cardiac troponin T assay was used with a limit of blank of 3 ng/L and limit of detection of 5 ng/L.^25^ The sex-specific 99th percentile URLs used in the current study were 9 ng/L and 16 ng/L in women and men, respectively.

### Suspected Macrotroponin Complexes

The presence of a suspected macrotroponin was defined a priori as a value above the sex-specific 99th percentile URL of cardiac troponin I or T where significant discordance (more than a 3-fold difference in absolute concentration) was observed between these assays.^16^ Participants were categorized into 3 groups: (1) no myocardial injury (those with cardiac troponin values above the limit of blank but within the normal reference range), (2) suspected macrotroponin (those with cardiac troponin concentrations above the sex-specific 99th percentile URL with assay discordance), and (3) myocardial injury (those with cardiac troponin concentrations above the sex-specific 99th percentile URL without assay discordance). In addition, individuals with both cardiac troponin I and cardiac troponin T concentrations above their respective sex-specific 99th percentile URLs were classified as myocardial injury.

### Clinical Outcomes

In GS:SFHS, follow-up data were available until July 2021. Linkage to National Health Service records via Scotland’s electronic Data Research and Innovation Service was used to obtain clinical outcome data, and cause of death data were obtained using the National Health Service Central Register. Cause-specific deaths and nonfatal cardiovascular events were classified using the 10^th^ revision of the *International Classification of Diseases, Tenth Revision* (*ICD-10*). In GS:SFHS, the primary outcome was a composite of major adverse cardiovascular events, including nonfatal myocardial infarction (I21, I22), nonfatal ischemic stroke or transient ischemic attack (I63, I64, G45), and cardiovascular death (all codes between I00 and I99). Secondary outcomes were the individual components of the composite outcome; namely, nonfatal myocardial infarction (I21, I22), nonfatal ischemic stroke or transient ischemic attack (I63, I64, G45), cardiovascular death (all codes between I00 and I99), a composite including heart failure hospitalization (I50, I11, I42.0, I42.6, I42.7, I42.9) and the components of the primary outcome, as well as all-cause death.

In NHANES, follow-up data were available until the end of December 2019. The vital status of participants was ascertained through a probabilistic match between NHANES personal identifiers and linkage to death certificates from the National Death Index.^26^ Cause-specific deaths were classified using *ICD-10*. In this cohort, the primary outcome was cardiovascular death (I00–I09, I11, I13, I20–I51, I60–I69), as data on nonfatal cardiovascular events were unavailable.

### Laboratory Methods and Measurements

To investigate the mechanisms of assay discordance and the proportion attributable to macrotroponin complexes, 2 complementary methods recommended by the International Federation of Clinical Chemistry were applied at Sahlgrenska University Hospital (Göteborg, Sweden) to a subset of samples from the GS:SFHS cohort study: ultracentrifugation and immunoglobulin depletion.^19^ Only samples from participants who had provided consent for their transfer outside the United Kingdom were included. All available samples from individuals with cardiac troponin I concentrations above the 99th percentile URL, both with and without assay discordance, were evaluated. Among individuals with cardiac troponin T above the 99th percentile URL, the 55 participants with the greatest assay discordance were selected and matched on the basis of cardiac troponin T concentration (±5 ng/L) to 55 participants with elevated cardiac troponin T but no discordance.

Ultracentrifugation and immunoglobulin depletion rely on different principles, one on molecular weight separation and the other on the specific binding of immunoglobulins. When both methods yield concordant results, macrotroponin complexes are reliably identified.^16,19^ Briefly, ultracentrifugation separates plasma proteins by apparent molecular mass: free cardiac troponin is approximately 20 to 40 kDa, whereas macrotroponin complexes are around 150 kDa. Under high centrifugal force, macrotroponin complexes sediment more rapidly toward the bottom of the tube than free cardiac troponin due to their higher molecular weight. Following ultracentrifugation, cardiac troponin concentrations are measured in the top and bottom fractions, and the relative distribution between these fractions allows differentiation of macrotroponin complexes from free troponin. For the immunoglobulin depletion method, cardiac troponin I (Alinity iSTAT high-sensitivity troponin I assay, Abbott Diagnostics) and cardiac troponin T (Elecsys Troponin T high-sensitivity assay, Roche Diagnostics) concentrations were remeasured using the Alinity i and Cobas e801 analyzers, respectively. The samples were then incubated with protein G–coated columns to capture immunoglobulin-bound proteins. The flow-through, containing unbound troponin, was collected, and cardiac troponin concentrations were remeasured on the same analyzers. A significant reduction (60%) in troponin levels following immunoglobulin depletion indicated the presence of macrotroponin complexes.^20^ A more detailed description of the ultracentrifugation and immunoglobulin methods is provided in the Supplemental Material.

### Statistical Analysis

Continuous variables were presented as mean±SD or median (25th–75th percentile), as appropriate. Categorical variables were presented as counts and percentages. Baseline characteristics were presented by cohort and groups.

For each group, we estimated the cumulative incidence of the primary outcome at 10 years of follow-up while considering death from noncardiovascular causes as competing risk, and group comparisons were made using Gray’s test.

Cause-specific Cox proportional hazards models were used to compare the risk of major adverse cardiovascular events in those with elevated cardiac troponin concentrations on either assay with and without a suspected macrotroponin. Death from noncardiovascular causes was considered a competing risk. Participants with cardiac troponin values above the limit of blank and within the normal reference range were used as the referent group. Models were adjusted for age and sex (model 1) and for age, sex, cardiovascular disease, cholesterol, high-density lipoprotein, systolic blood pressure, smoking status, family history, diabetes, rheumatoid arthritis, social deprivation, and lipid-modifying and antihypertensive medication (model 2). Results are presented as the hazard ratio (HR) and 95% CI. The same approach was used to examine the associations of macrotroponin groups with secondary outcomes in GS:SFHS.

In addition, we performed several sensitivity analyses. First, we applied a more stringent threshold for assay discordance, using a 5-fold difference criterion instead of a 3-fold difference. Second, we examined the impact of excluding individuals with a history of cardiovascular disease at baseline. Third, we assessed the associations of macrotroponin groups with cardiovascular events, using individuals with cardiac troponin I or T concentrations above the limit of detection, rather than the limit of blank, as the reference group.

Last, we assessed the generalizability of our findings by evaluating the frequency of suspected macrotroponin complex formation identified through assay discordance in the NHANES cohort. Participants were stratified into the same groups as for the GS:SFHS, and the association between group and risk of cardiovascular death was evaluated using the same approach as in the primary analysis for GS:SFHS. Survey weights were applied to ensure that estimates were representative of the 1999 to 2004 US adult population.

Single imputation was applied for covariates, using predictive mean matching or logistic regression as appropriate. Statistical analysis was performed in R version 4.3.2. The original R code used in these analyses is available upon request from the corresponding author.

## RESULTS

In GS:SFHS, 19 499 participants had measurements available for both cardiac troponin I and cardiac troponin T (Figure S1). Their median age at enrollment was 49 (25th–75th percentile, 36–58) years, and 58.3% of participants were women (Table S1). A history of cardiovascular disease, hypertension, and diabetes was self-reported in 4.5%, 8.1%, and 2.9% of participants, respectively. In total, 14 578 (75%) and 10 395 (53%) had cardiac troponin concentrations above the limit of blank of the cardiac troponin I and T assays, respectively.

### Discordance and Macrotroponin Complex Formation

For cardiac troponin I, values were above the sex-specific URL in 193 of 19 499 (1%) participants, with significant assay discordance occurring in 93 (48%) of these participants (Figure 2A; Figure S2). For cardiac troponin T, values were above the sex-specific URL in 1 159 of 19 499 (6%) participants, with assay discordance occurring in 655 (57%) of these participants (Figure 2A). Participants with discordance due to a suspected macrotroponin I and T were older (56 [25th–75th percentile, 47–61] and 59 [48–66] years) than those with no myocardial injury on either assay (52 [40–60] and 53 [40–61] years). Risk factors and history of cardiovascular disease differed across the groups, with similar trends for cardiac troponin I and T where those with myocardial injury were more likely to have cardiovascular disease or diabetes (Table 1 and Table 2). A similar proportion of suspected macrotroponin was present in women and men and when stratified by age (Figure S3).

**Table 1.**
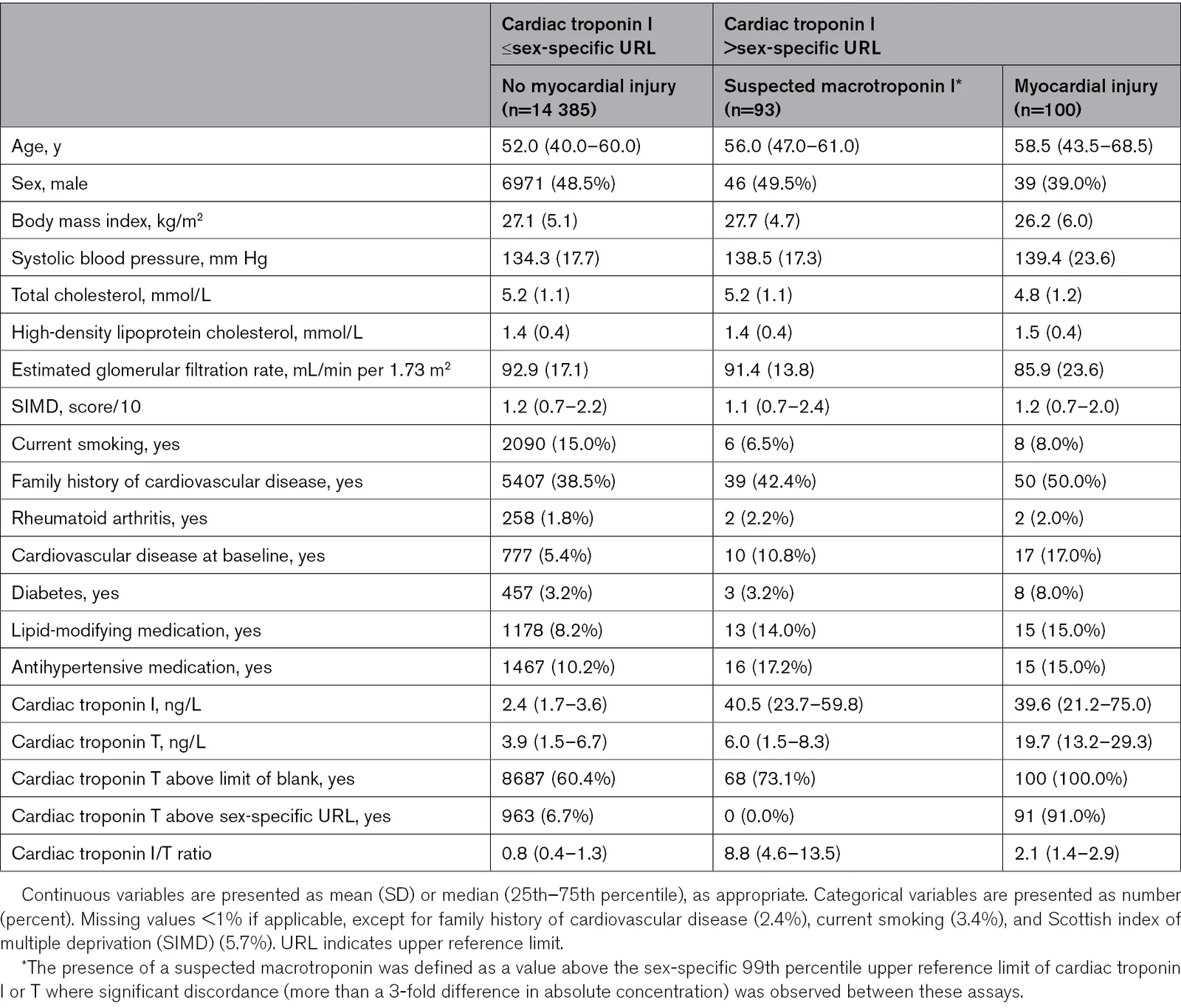
Baseline Characteristics of Participants With Cardiac Troponin I Concentrations Above the Limit of Blank

**Table 2.**
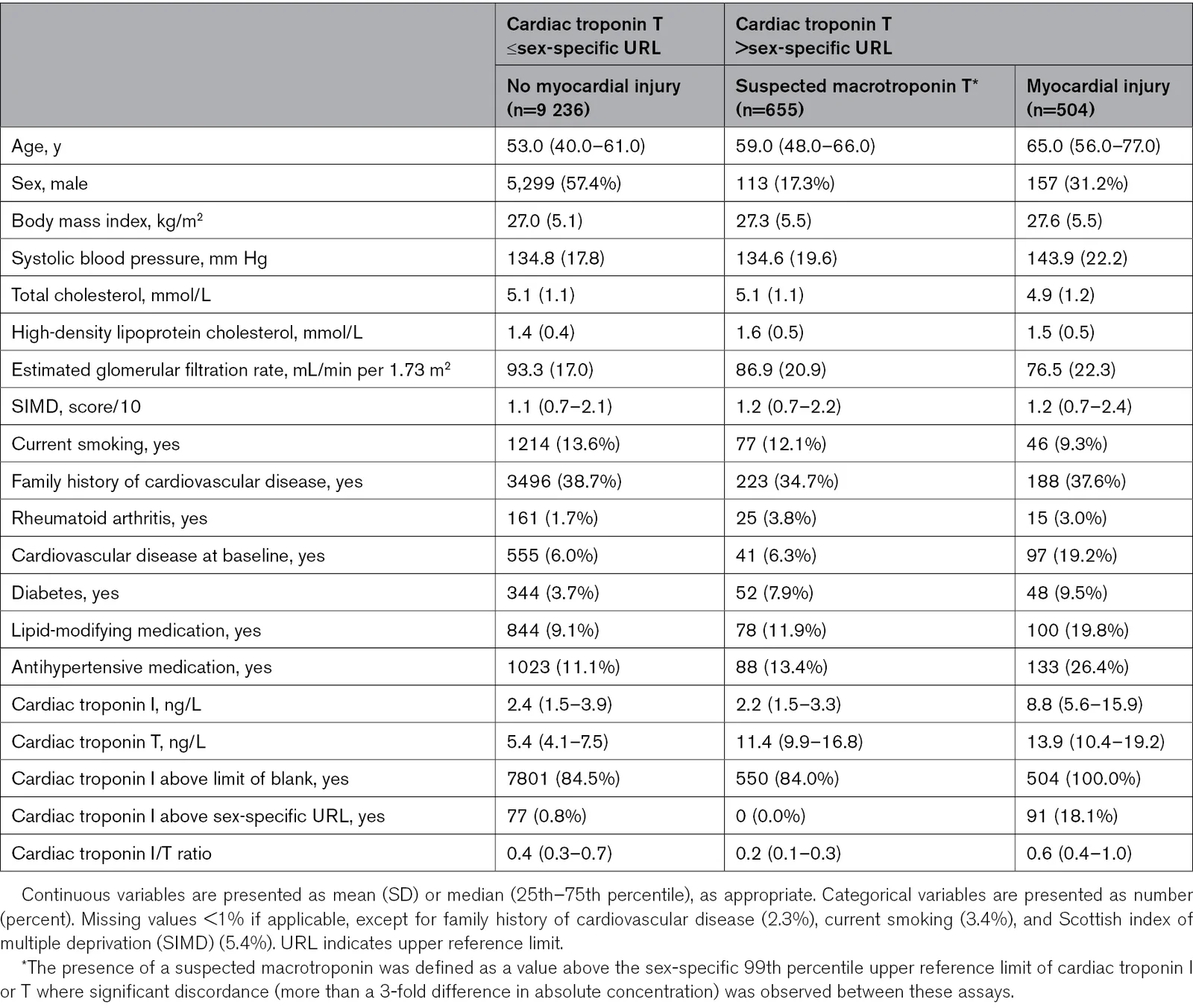
Baseline Characteristics of Participants With Cardiac Troponin T Concentrations Above the Limit of Blank

**Figure 2.**
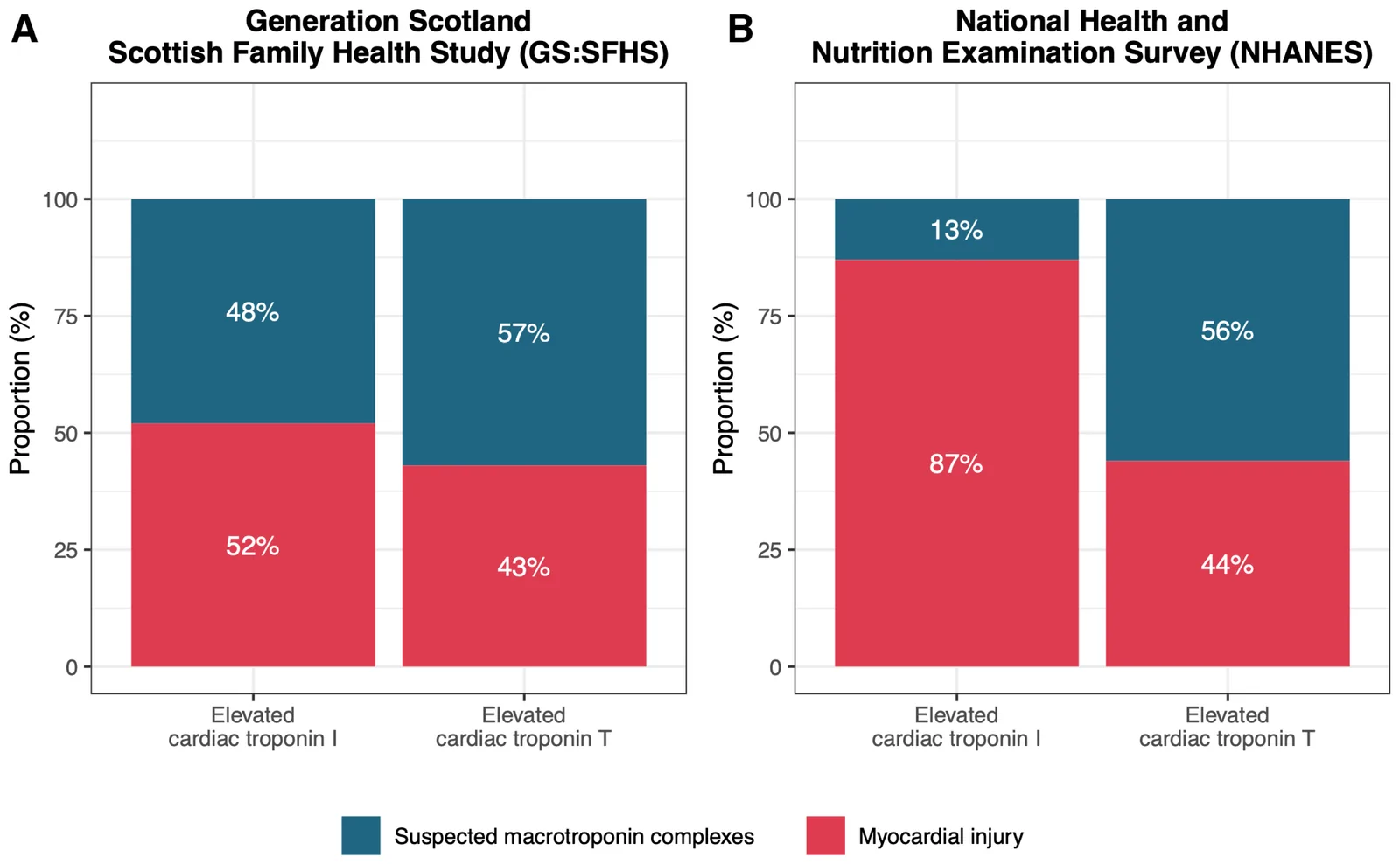
**Prevalence of suspected macrotroponin complexes in GS:SFHS (Generation Scotland Scottish Family Health Study) and NHANES (National Health and Nutrition Examination Survey) general population cohorts.** Shown is the proportion of individuals with suspected macrotroponin complexes or myocardial injury among those with elevated cardiac troponin levels in GS:SFHS (**A**) and NHANES (**B**). Suspected macrotroponin was defined as a value above the sex-specific 99th percentile upper reference limit of cardiac troponin I or T, where significant discordance (more than a 3-fold difference in absolute concentration) was observed between these assays.

A combination of ultracentrifugation and immunoglobulin depletion was used to identify macrotroponin complex formation in 82 of the 193 participants with an elevated cardiac troponin I concentration above the sex-specific URL (Figure 3; Figure S2). In the remainder, measurement was precluded, as consent was not provided to transfer samples outside the United Kingdom (n=100) or there was insufficient volume of stored plasma (n=11). Macrotroponin I complex was confirmed in 92.5% (49 of 53) of those with assay discordance and was identified in 41.4% (12 of 29) of those without discordance. Among the 1159 participants with elevated cardiac troponin T concentrations above the sex-specific URL, we selected those with suspected macrotroponin and the greatest assay discordance (n=55). These were matched on the basis of cardiac troponin T concentration (±5 ng/L) to participants with myocardial injury and no discordance (n=55). In the majority, it was not possible to measure macrotroponin complex formation, as the cardiac troponin T concentrations were insufficient for reliable measurement (n=76) or there was insufficient plasma volume (n=7). In the remainder, macrotroponin T complex was confirmed in 15.4% (2 of 13) of those with assay discordance and was also identified in 7.1% (1/14) of those without discordance.

**Figure 3.**
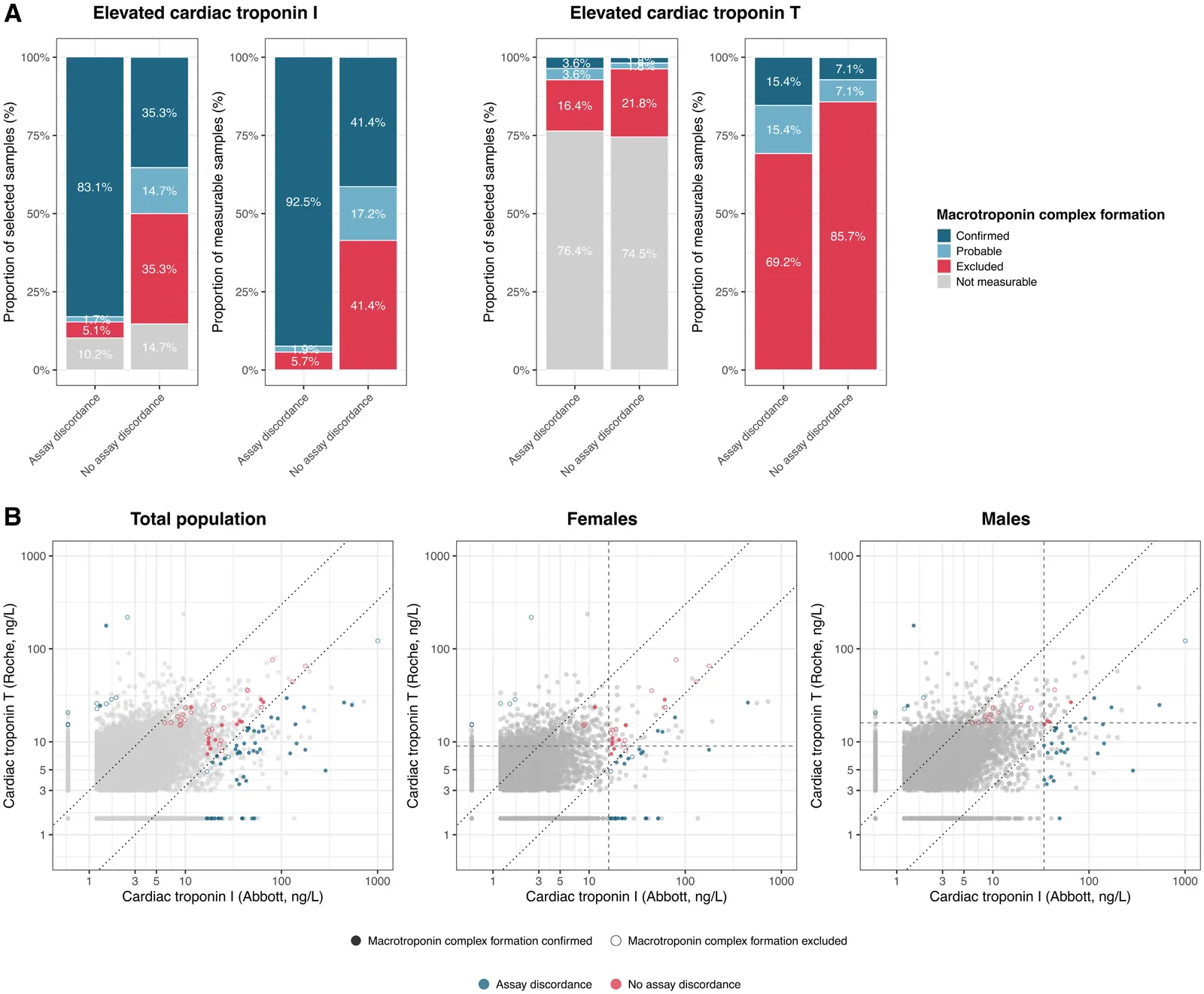
**Macrotroponin complex formation in participants with elevated cardiac troponin I and T concentrations with or without discordance. A**, Macrotroponin identification by ultracentrifugation and immunoglobulin depletion. **B**, Macrotroponin complex formation relative to cardiac troponin concentrations. Ultracentrifugation and immunoglobulin depletion were performed in all 93 participants with an elevated cardiac troponin I concentration above the sex-specific 99th percentile upper reference limit who had provided consent for sample transfer outside the United Kingdom and in 55 participants with suspected macrotroponin T complex formation and the greatest assay discordance matched on the basis of cardiac troponin T concentration (±5 ng/L) to 55 participants with presumed myocardial injury. When ultracentrifugation and immunoglobulin depletion yield concordant results, macrotroponin complexes are reliably identified and classified as confirmed or excluded; when the 2 methods are discordant, macrotroponin complexes are considered probable.

### Macrotroponin Complex and Risk of Major Adverse Cardiovascular Events

For participants with a cardiac troponin I concentration above the limit of blank, the incidence of major adverse cardiovascular events at 10 years was 6% (845 of 14 385) in those without myocardial injury (referent), 13% (12 of 93) in those with suspected macrotroponin complex, and 28% (28 of 100) in participants with myocardial injury (Figure 4A; Figure S4). Compared with those without myocardial injury (the referent group), participants with myocardial injury had an increased risk of major adverse cardiovascular events (unadjusted HR, 4.69 [95% CI, 3.25–6.79]). This increase in risk persisted after adjustment for age and sex (model 1; adjusted HR, 3.06 [95% CI, 2.11–4.45]) and for age, sex, cardiovascular disease, and risk factors (model 2; adjusted HR, 2.90 [95% CI, 1.99–4.22]) (*P*<0.001 for both; Table 3). Those with a suspected macrotroponin were also at increased risk of major adverse cardiovascular events compared with the referent group (unadjusted HR, 2.02 [95% CI, 1.19–3.42]; *P*=0.009), but the association was attenuated and no longer statistically significant following adjustment for age and sex (model 1; adjusted HR, 1.69 [95% CI, 1.00–2.87]; *P*=0.050) and additional risk factors (model 2; adjusted HR, 1.59 [95% CI, 0.94–2.70]; *P*=0.086) (Table 3).

**Table 3.**
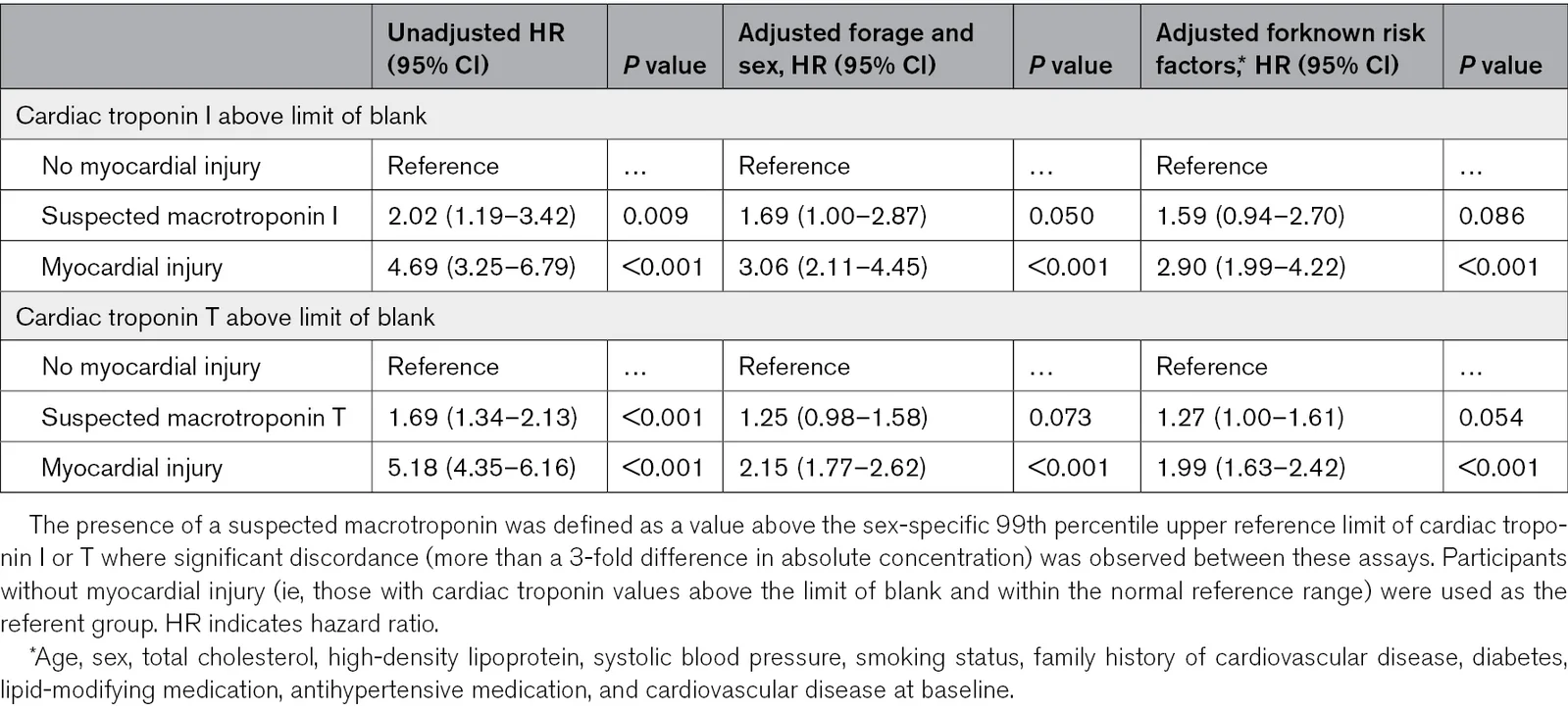
Suspected Macrotroponin Complexes and Risk of Major Cardiovascular Adverse Events

**Figure 4.**
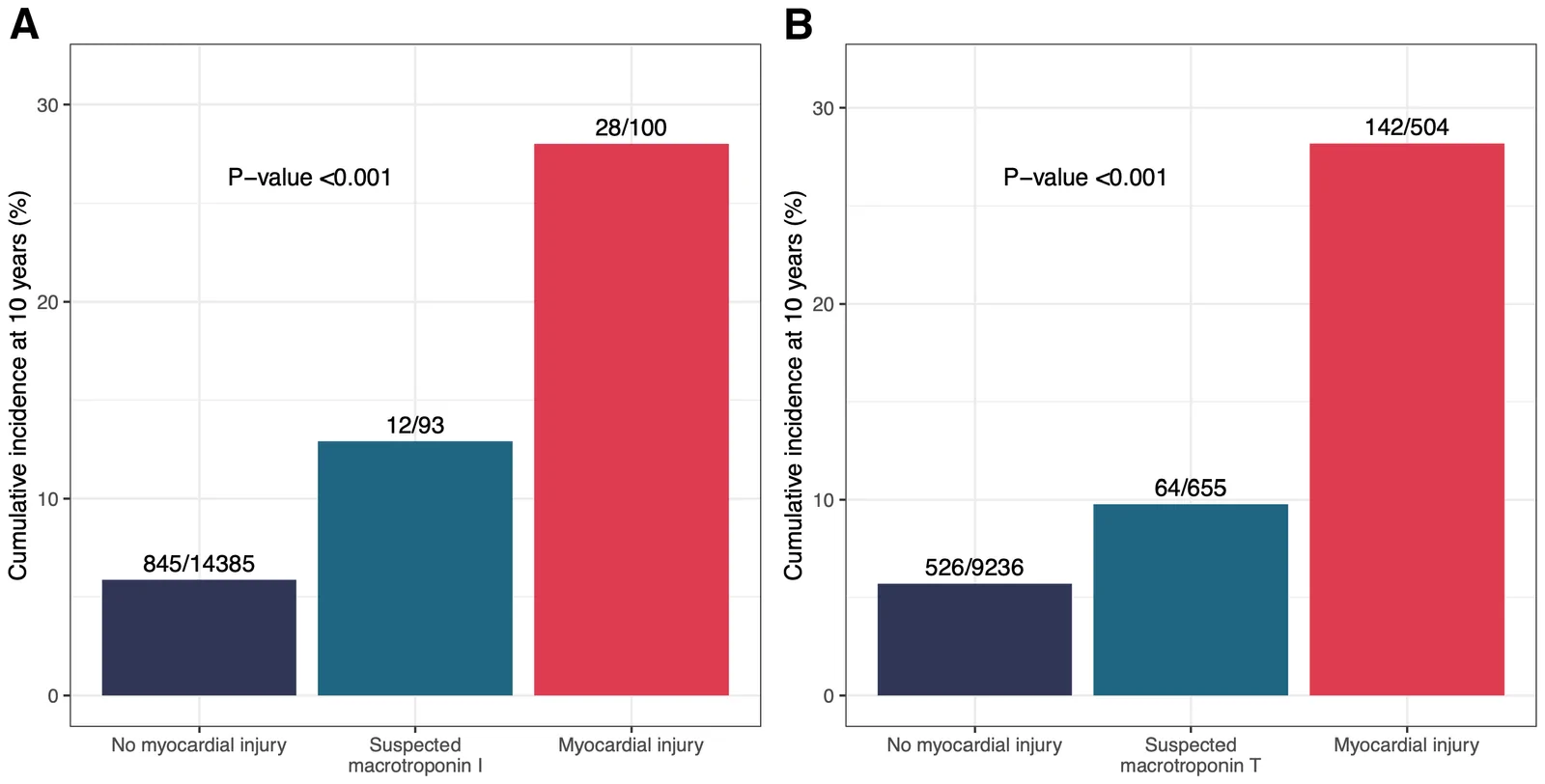
**Cumulative incidence of major adverse cardiovascular events at 10 years in participants with and without suspected macrotroponin I and T complexes. A**, Macrotroponin I. **B**, Macrotroponin T. The presence of a suspected macrotroponin was defined as a value above the sex-specific 99th percentile upper reference limit of cardiac troponin I or T, where significant discordance (more than a 3-fold difference in absolute concentration) was observed between these assays. Major adverse cardiovascular events included nonfatal myocardial infarction, nonfatal ischemic stroke or transient ischemic attack, or cardiovascular death.

In participants with a cardiac troponin T concentration above the limit of blank, the incidence of major adverse cardiovascular events at 10 years was 6% (526 of 9236) in those without myocardial injury (referent), 10% (64 of 655) in those with suspected macrotroponin complex, and 28% (142 of 504) in those with myocardial injury (Figure 4B; Figure S4). Compared with those without myocardial injury (the referent group), participants with myocardial injury were at increased risk of major adverse cardiovascular events (unadjusted HR, 5.18 [95% CI, 4.35–6.16]), which persisted following adjustment (model 1: adjusted HR, 2.15 [95% CI, 1.77–2.62]; model 2: adjusted HR, 1.99 [95% CI, 1.63–2.42]; *P*<0.001 for both; Table 3). While those with suspected macrotroponin also appeared to be at higher risk compared with the referent group (unadjusted HR, 1.69 [95% CI, 1.34–2.13]; *P*<0.001), this was no longer statistically significant following adjustment for age and sex (model 1; adjusted HR, 1.25 [95% CI, 0.98–1.58]; *P*=0.073) and additional risk factors (model 2; adjusted HR, 1.27 [95% CI, 1.00–1.61]; *P*=0.054; Table 3).

### Macrotroponin Complex and Risk of Secondary Outcomes

The cumulative incidence rates for secondary outcomes at 10 years are shown in Figure S5. After adjusting for age, sex, and additional risk factors, no statistically significant associations were found between suspected macrotroponin I complexes and the risk of nonfatal myocardial infarction, nonfatal stroke, or cardiovascular death (Table S2). Similarly, no statistically significant associations were observed for suspected macrotroponin T complexes with nonfatal myocardial infarction or nonfatal stroke. However, compared with individuals with cardiac troponin T concentrations within the normal reference range, those with suspected macrotroponin T complexes had an increased risk of cardiovascular death following adjustment for age, sex, and cardiovascular risk factors (model 2; adjusted HR, 1.58 [95% CI, 1.16–2.15]; *P*=0.004). A similar association was observed when hospitalization for heart failure was included in the composite end point (Figure S6 Table S3) or when evaluating all-cause death (Figure S7; Table S4).

### Sensitivity Analysis

When using a stricter definition of discordance (5-fold difference), 34% (66 of 193) of participants with elevated troponin I values and 33% (383 of 1159) with elevated troponin T values were identified as having a suspected macrotroponin complex. The associations with major cardiovascular events remained consistent with the primary analysis (Figure S8; Table S5), except that the association for those with suspected macrotroponin T complexes remained significant after adjustment for known cardiovascular risk factors. A similar pattern was observed when excluding participants with known cardiovascular disease at baseline (Figure S9; Table S6). When restricting the analysis to those with cardiac troponin concentrations above the limit of detection rather than the limit of blank, the findings were consistent with those in the main analysis (Figure S10; Table S7).

### National Health and Nutrition Examination Survey

In NHANES, 13 652 participants had a measurement of both cardiac troponin assays available (Figure S1; Table S1), with 9737 (71%) and 11 784 (86%) having concentrations above the limit of blank of the cardiac troponin I and T assays, respectively. For cardiac troponin I, values were above the sex-specific URL in 225 of 9 737 (2%) participants, with significant assay discordance occurring in 27 (12%) of these participants (Table S8). For cardiac troponin T, values were above the sex-specific URL in 1982 of 11 784 (17%) participants, with assay discordance occurring in 1089 (55%) of these participants (Table S9). The prevalence estimate (number of US adults) of an elevated cardiac troponin I was 1% (2.4 million), with discordance occurring in 13% (0.3 million) (Figure 2B; Table S10). The prevalence estimate of an elevated cardiac troponin T was 11% (21.6 million), with discordance occurring in 56% (12.1 million) (Figure 2B; Table S11). In NHANES, elevated cardiac troponin I values were more likely to be discordant in men and younger participants, whereas elevated cardiac troponin T values showed similar rates of discordance across sexes and when stratified by age (Figure S11).

After adjusting for known cardiovascular risk factors, participants with an elevated cardiac troponin I or cardiac troponin T concentration without discordance had an increased risk of cardiovascular death during follow-up (Table S12). In contrast, no significant association with cardiovascular death was observed in those with suspected macrotroponin I complexes (model 2; adjusted HR, 0.67 [95% CI, 0.08–5.31]; *P*=0.706; Table S12). Participants with an elevated cardiac troponin T concentration and discordance remained at higher risk of cardiovascular death (model 2; adjusted HR, 1.80 [95% CI, 1.44–2.25]; *P*<0.001; Table S8).

Discordance was quantified using an additional cardiac troponin I assay in the replication cohort (Figure S12), demonstrating that the frequency of suspected macrotroponin complex varies depending on the assay pairing.

## DISCUSSION

We evaluated the frequency of suspected macrotroponin complex formation in 2 general population cohorts and determined whether this was associated with future cardiovascular events. There are 4 main findings with implications for clinical practice. First, approximately half of those found to have an elevated cardiac troponin I or T concentration in the general population have discordance between assays that may be a consequence of assay interference. Second, the risk of major adverse cardiovascular events in those with an elevated cardiac troponin and significant discordance was comparable with those without evidence of myocardial injury on either assay. In contrast, elevated cardiac troponin concentrations without discordance, suggestive of myocardial injury, were associated with a 2- to 3-fold increase in the risk of major adverse cardiovascular events independent of age, sex, and known cardiovascular disease or risk factors. Third, macrotroponin I complexes were confirmed in 9 of 10 individuals with an elevated cardiac troponin I concentration and discordance and in 4 of 10 individuals with an elevated cardiac troponin I without discordance. Finally, macrotroponin T complexes appeared to be less frequent despite a far greater proportion of the population having elevated cardiac troponin T values and similar levels of assay discordance. Together, our findings suggest that assay interference due to macrotroponin I complex formation is common and could have important clinical implications for risk stratification with cardiac troponin I in the general population. In contrast, macrotroponin T complex formation appears to be a less likely explanation for elevated cardiac troponin T levels in the general population despite similar levels of discordance.

Cardiac troponin is detectable in the circulation of healthy persons, with levels varying on a daily basis and increasing in response to physical exercise.^27,28^ The chemiluminescence immunoassays that are used to quantify cardiac troponin are prone to interference,^19^ where antibodies can bind with cardiac troponin fragments to form complexes in circulation (Figure 1). These macrotroponin complexes have a longer half-life than unbound cardiac troponin and accumulate in the circulation due to reduced clearance rather than acute or chronic myocardial injury,^14^ with the potential to create uncertainty for clinicians and cause anxiety for patients.^15^ Little is known about the prior exposures that lead to the development of antibodies responsible for macrotroponin complex formation, but they appear to be more common in individuals with prior myocardial infarction or established cardiovascular disease.^29^ Despite numerous case reports of macrotroponin complex formation in patients with possible myocardial infarction or acute myocarditis in whom the diagnosis was excluded on coronary and cardiac imaging,^30–32^ few studies have systematically evaluated the role of interference in the general population. Systematic studies are important to guide interpretation, as cardiac troponin testing is increasingly performed to assess risk in asymptomatic persons or in patients with stable symptoms due to chronic coronary artery disease or valvular heart disease.^33,34^

Our findings are consistent with those from a study of patients with an elevated cardiac troponin I concentration where testing was performed in a community setting.^35^ Hospital admission was arranged for further investigation, and 53% (99 of 188) of elevated values were found to be a consequence of macrotroponin complex formation. Those with macrotroponin complexes were less likely to be diagnosed with acute coronary syndrome, had longer lengths of stay to complete investigations, and were less likely to have died during follow-up. Macrotroponin complex formation was recently quantified in a cohort of 1058 healthy individuals participating in a healthy reference range study for cardiac troponin. Here, macrotroponin complex was found in 76% of participants with an elevated cardiac troponin I value; excluding these individuals substantially reduced the estimated sex-specific 99th percentile URL recommended for the diagnosis of myocardial infarction.^20^ Consistent with our findings, in a cohort of 159 hospitalized patients with elevated cardiac troponin I concentrations, probable macrotroponin identified by polyethylene glycol precipitation was associated with a lower risk of long-term all-cause and cardiovascular mortality compared with troponin elevations without evidence of macrotroponin.^36^ While cardiac troponin I/T ratios have been evaluated as a predictor of cardiovascular disease risk,^37^ to our knowledge, no previous studies have evaluated the impact of assay discordance and macrotroponin complex formation on cardiovascular disease risk prediction in the general population.

Our findings from 2 large general population cohorts in Europe and the United States suggest that half of elevated cardiac troponin I concentrations in apparently healthy individuals may be a consequence of assay interference. Using gold-standard methodology, we determined that an elevated cardiac troponin I concentration and significant discordance were due to macrotroponin complex formation in 9 of 10 individuals. Furthermore, 4 of 10 individuals with elevated cardiac troponin I concentrations without discordance were also found to have macrotroponin complex formation, suggesting that the recommended approach of using a >3-fold difference between cardiac troponin I and T concentrations may not be sufficiently sensitive to identify all of those with macrotroponin complex formation.^16^ While cardiac troponin T concentrations were elevated in 6 times as many individuals, only in 3 of 110 samples tested was this confirmed to be due to macrotroponin T complex formation. The higher frequency of elevated cardiac troponin T concentration may be attributed to aging and declining renal function, both of which have a greater influence on cardiac troponin T concentration than on cardiac troponin I.^38^ Elevated cardiac troponin T concentration may also be attributable to subclinical skeletal muscle pathology, as elevation has been reported in patients with skeletal myopathies in the absence of overt cardiac disease.^39,40^ These comorbid conditions could explain the residual association between suspected macrotroponin T and secondary outcomes, such as cardiovascular death and heart failure hospitalization, following adjustment for traditional cardiovascular risk factors. An alternative explanation is that this group may include a higher proportion of individuals with true subclinical myocardial injury, as the proportion of elevated cardiac troponin T values with discordance attributable to macrotroponin formation may be smaller in this population, and a >3-fold difference between cardiac troponin T and I was less predictive of macrotroponin complexes. Our analysis did not result in a more effective approach to the identification of assay discordance, with similar findings observed when using a 3-fold and 5-fold difference. Further research is needed to develop more robust approaches for identifying individuals with an elevated cardiac troponin concentration attributable to macrotroponin complex formation, beyond the current crude measure of discordance, which appears to identify macrotroponin I more effectively than macrotroponin T.^41^

Importantly, those identified as having a suspected macrotroponin were at comparable risk of major adverse cardiovascular events as those without myocardial injury in both cohorts with over 15 years of follow-up. These findings are important and have clinical implications for the use of high-sensitivity cardiac troponin assays for risk stratification in the general population. Addition of cardiac troponin measurement to conventional risk prediction models can improve discrimination for future cardiovascular events.^11,42^ Given that the NHANES cohort is representative of 205 million people in the United States, with a similar age distribution, population-wide screening with cardiac troponin I could potentially identify around 320 000 individuals where elevated values are due to macrotroponin complex formation rather than myocardial injury due to subclinical cardiovascular disease. Without knowledge of the frequency of cardiac troponin interference in the general population, the widespread use of cardiac troponin testing for cardiovascular risk assessment could result in unnecessary cardiac investigation and prescribing due to incorrect cardiovascular risk assessment. Given that methods for detecting macrotroponin complex formation are not available in most clinical laboratories, we would recommend that all asymptomatic persons with an elevated cardiac troponin concentration have measurement repeated using an alternative cardiac troponin assay or have samples referred for macrotroponin analysis at a specialized laboratory before considering cardiac imaging for the presence of subclinical cardiovascular disease. Recognition of assay interference may improve the utility of cardiac troponin for risk stratification in population settings.

We evaluated the prevalence and associations of assay interference due to macrotroponin complex formation in a general population of asymptomatic individuals. The prevalence is likely to be similar in symptomatic patients, but identifying those with interference is more challenging, given that the majority of elevated values in this setting are due to acute or chronic myocardial injury. Systematic studies are now required in symptomatic patients with persistently elevated cardiac troponin values to determine what proportion is due to chronic myocardial injury and underlying structural heart disease and what proportion is due to macrotroponin complex formation.

Our study has several strengths. First, this is the first systematic investigation to evaluate the frequency of macrotroponin complexes identified through cardiac troponin assay discordance in a general population setting. So far, studies have largely been limited to case reports, selected clinical populations, or laboratory-based investigations, limiting the generalizability of the findings. Second, the availability of complete and long-term follow-up in both Scotland (over 15 years) and the United States (over 20 years) provided sufficient numbers of cardiovascular events to assess the association between assay discordance and future risk of major adverse cardiovascular events. Third, the confirmation of macrotroponin complexes using molecular weight assessment by ultracentrifugation together with immunoglobulin depletion in a subset of discordant samples strengthens the biological plausibility of our findings by directly demonstrating the mechanism underlying assay interference. Finally, the use of two independent population-based cohorts enhances the robustness and external validity of our results.

Some limitations also need to be acknowledged. First, although the previously suggested 3-fold difference threshold appears to be effective for ruling in macrotroponin I complex formation (9 of 10 participants with elevated cardiac troponin I and assay discordance had confirmed macrotroponin complexes), our findings suggest that it may be less reliable for ruling out macrotroponin I, as 4 of 10 participants without assay discordance also had confirmed complexes. Conversely, the majority of individuals with elevated cardiac troponin T and 3-fold discordance were not found to have macrotroponin complexes, which may account for the residual association between suspected macrotroponin T and cardiovascular death. Assay discordance is unlikely to identify samples containing macrotroponin complexes or autoantibodies binding both cardiac troponin T and I subunits, but these are likely less frequent than those affecting individual subunits. Further research is needed to evaluate alternative approaches for suspected macrotroponin, particularly when this is identified using a cardiac troponin T assay, including machine learning or other model-based methods.^17^ Additionally, while we used state-of-the-art methods to confirm or exclude macrotroponin complex formation as an explanation for assay discordance, these methods do not have sufficient precision to detect small cardiac troponin T elevations due to macrotroponin complexes near the URL. In the current study, it was only feasible to verify macrotroponins in a subset of participants with elevated cardiac troponin T, as more than 1 in 20 individuals had elevated concentrations, and over 600 exhibited marked discordance. A priori, we selected those with the greatest discordance and highest cardiac troponin T concentrations, but despite this, the concentrations were insufficient to confirm or exclude a macrotroponin complex in three-quarters of those tested. Furthermore, we only evaluated the frequency of macrotroponin complex formation for 1 high-sensitivity cardiac troponin I assay, but there are multiple assays available and used routinely in clinical practice. This is an important limitation of our study, as it is likely that the frequency of elevated cardiac troponin concentrations due to interference varies by assay.^43^ To illustrate this, we explored prevalence estimates using alternative assay-to-assay discordance patterns in NHANES, highlighting that estimates of macrotroponin complex formation identified through discordance are assay specific and may not be directly generalizable across different clinical platforms. The frequency of interference is also likely to vary according to the characteristics of the population, with a greater proportion of elevated cardiac troponin concentrations attributable to macrotroponin complex formation in lower-risk populations with a lower burden of subclinical cardiovascular disease. Our observations on the association between suspected macrotroponin complex formation and future risk of cardiovascular death were replicated in the NHANES cohort, but data on the nonfatal myocardial infarction and stroke components of the composite primary outcome were not available. Finally, it should be recognized that the present analyses are based on an earlier NHANES survey cycles (1999–2004). The sampling framework of these cycles may not fully reflect the current US general population, which should be considered when interpreting the generalizability of our findings.

In conclusion, half of all elevated cardiac troponin I or T concentrations are discordant in the general population and may be due to assay interference. Macrotroponin complex formation was responsible for the majority of elevated cardiac troponin I concentrations but not cardiac troponin T elevations despite similar levels of discordance. Importantly, individuals with an elevated cardiac troponin and discordance were at comparable risk of cardiovascular events as those without myocardial injury. Together, these findings have important implications for the use of cardiac troponin testing for cardiovascular risk assessment in the general population.

## ARTICLE INFORMATION

### Author Contributions

Dr de Bakker, Dr Hammarsten, Dr Eggers, Dr Lindahl, and Dr Mills conceived the study and its design. Dr de Bakker, Dr Hammarsten, and Dr Khachatryan performed the analysis. Dr de Bakker, Dr Hammarsten, Dr Eggers, Dr Lindahl, Dr Omland, Dr Welsh, and Dr Mills interpreted the data. Dr de Bakker, Dr Thurston, and Dr Mills drafted the manuscript. Dr de Bakker, Dr Thurston, Dr Hammarsten, Dr Khachatryan, Dr Fujisawa, Dr Li, Dr Eggers, Dr McEvoy, Dr Lindahl, Dr Omland, Dr Sattar, Dr Sudlow, Dr Welsh, and Dr Mills revised the manuscript critically for important intellectual content. All authors provided their final approval of the version to be published. All authors are accountable for the work.

### Sources of Funding

Generation Scotland received funding from the Chief Scientist Office of the Scottish Government Health Directorates (CZD/16/6) and the Scottish Funding Council (HR03006) and is currently supported by the Wellcome Trust (216767/Z/19/Z). Dr de Bakker and Dr Thurston are supported by an intermediate basic science research fellowship (FS/IBSRF/23/25161), research excellence award (RE/24/130012), and clinical research training fellowship (FS/CRTF/25/24735) from the British Heart Foundation, respectively. Dr Li is supported by a project grant (PG/25/12298) from the British Heart Foundation. Dr Mills is supported by a chair award (CH/F/21/90010), a program grant (RG/F/25/110169), and a research excellence award (RE/24/130012) from the British Heart Foundation.

### Disclosures

Dr Thurston has received honoraria from Roche Diagnostics. Dr Hammarsten has received honoraria from Siemens Healthineers and LumiraDx and holds stock and stock options from AlignedBio. Dr Lindahl has a grant from the Swedish Heart and Lung Foundation unrelated to the submitted work. Dr Omland has received honoraria from Abbott Diagnostics, NovoNordisk, CardiNor, and Roche Diagnostics and is employed by Akershus University Hospital, which has received research funding from Abbott Diagnostics, ChromaDex, Novartis, and Roche Diagnostics. Dr Sattar has consulted for or received honoraria from Abbott Laboratories, AbbVie, Amgen, AstraZeneca, Boehringer Ingelheim, Eli Lilly, Hanmi Pharmaceuticals, Janssen, Menarini-Ricerche, Merck Sharp & Dohme, Novartis, Novo Nordisk, Pfizer, Roche Diagnostics, and Sanofi and received grant support paid to his University from AstraZeneca, Boehringer Ingelheim, Novartis, and Roche Diagnostics outside the submitted work. Dr Eggers has received consultancy fees from Roche Diagnostics. Dr Welsh has received a research grant from Roche Diagnostics outside of the submitted work as well as grant income from AstraZeneca, Boehringer Ingelheim, and Novartis outside the submitted work. Dr Mills has received honoraria from Abbott Laboratories, Siemens Healthineers, and Roche Diagnostics over the last 3 years.

### Supplemental Material

Supplemental Methods

Tables S1–S12

Figures S1–S12

STROBE checklist

## Supplementary Material
