# Supplementary material for: Macrotroponin Complexes and Risk of Cardiovascular Events
Source: Circulation. 2026 Sep 8;154(18):1777–90. doi: 10.1161/CIRCULATIONAHA.126.080483 (PMC13722435; doi:10.1161/CIRCULATIONAHA.126.080483)
Supplement: Supplementary file 1 [file cir-154-1777-s001.pdf]

SUPPLEMENTAL MATERIAL

Macrotroponin complexes and risk of cardiovascular events

Running title: Macrotroponin and cardiovascular events

Marie de Bakker, PhD,[1,2*] Alexander JF Thurston, MD,[1*] Ola Hammarsten, MD, PhD,[3]
Mareta Khachatryan, BSc,[3] Takeshi Fujisawa, PhD,[1,4] Ziwen Li, PhD,[1]
John W McEvoy, MB BCh, PhD,[5,6,7] Bertil Lindahl, MD, PhD,[8]
Torbjørn Omland, MD, PhD, MPH,[9,10] Naveed Sattar, MB BCh, PhD,[11]
Cathie LM Sudlow, MD, PhD,[2] Kai M Eggers, MD, PhD,[8]
Paul Welsh, PhD,[11*] Nicholas L Mills, MD, PhD[1*]

[1]Institute for Neurosciences and Cardiovascular Research, University of Edinburgh, Edinburgh, United Kingdom
[2]Usher Institute, University of Edinburgh, Edinburgh, United Kingdom
[3]Department of Clinical Chemistry, Sahlgrenska University Hospital, Göteborg, Sweden
[4]British Heart Foundation Cardiovascular Biomarker Laboratory, University of Edinburgh, Edinburgh, United Kingdom
[5]National Institute for Prevention and Cardiovascular Health and University of Galway School of Medicine, Galway, Ireland
[6]Division of Cardiology, Johns Hopkins School of Medicine, Johns Hopkins University, Baltimore, Maryland, United States
[7]Department of Epidemiology and the Welch Center for Prevention, Epidemiology, and Clinical Research, Johns Hopkins Bloomberg School of Public Health, Baltimore, Maryland, United States
[8]Department of Medical Sciences and Uppsala Clinical Research Center, Uppsala University, Uppsala, Sweden
[9]Department of Cardiology, Akershus University Hospital, Lørenskog, Norway
[10]K.G. Jebsen Centre for Cardiac Biomarkers, University of Oslo, Oslo, Norway
[11]School of Cardiovascular & Metabolic Health, University of Glasgow, Glasgow, United Kingdom

* Contributed equally

Supplemental Methods

Detection of macrotroponin complexes by ultracentrifugation and immunodepletion

High–molecular weight troponin complexes were identified using two complementary and independent approaches: size-based separation using density-assisted ultracentrifugation and IgG immunodepletion with Protein G. This strategy distinguishes free cardiac troponin from immunoglobulin-bound macrotroponin complexes based on molecular size and IgG depletion.

For the size-based separation, serum samples were centrifuged (18,840 × g, 10 min), diluted in TBS-C (20 mM Tris-HCl, pH 7.5; 150 mM NaCl; 2.5 mM $CaCl_2$), and layered onto a frozen 54% sucrose cushion prepared in 1.5 mL polypropylene tubes (Beckman Coulter). Samples were centrifuged at 390,900 × g (85,000 rpm) for 75 min at 20 °C using a TLA-100.3 rotor in a Beckman Coulter Optima Max-XP ultracentrifuge. Conditions were optimized to separate free cardiac troponin (~17–40 kDa) from macrotroponin complexes (>150 kDa). Following centrifugation, equal top and bottom fractions were collected and troponin concentrations were analyzed.

Cardiac troponin was quantified using either the Abbott Alinity i STAT High Sensitivity Troponin I assay (Abbott Diagnostics) or the Roche Elecsys® high-sensitivity cardiac Troponin T (hs-cTnT) assay on a cobas e 801 analyzer (Roche Diagnostics). The proportion of cardiac troponin in the upper fraction was expressed as the percentage of total recovered cardiac troponin. Samples were classified as macrotroponin I if <20% of total cardiac troponin I and as macrotroponin T if <30% of total cardiac troponin T was detected in the upper fraction following ultracentrifugation. Fractions were pooled and defined as input for immunodepletion.

IgG-associated complexes were removed using Protein G Sepharose 4 Fast Flow (GE Healthcare) packed into Mini Bio-Spin chromatography columns (Bio-Rad). The resin, supplied in TBS containing 25% ethanol, was equilibrated by centrifugation to remove storage buffer and subsequently washed with TBS (20 mM Tris-HCl, pH 7.5; 150 mM NaCl) prior to sample application.

Input samples were applied to the equilibrated Protein G resin and incubated for 1 min at room temperature. Flow-through (Protein G–treated sample) was collected by centrifugation and analyzed for cardiac troponin concentration. The concentration of cardiac troponin in the flow-through was compared with that in the corresponding input sample, and the reduction was calculated as the percentage decrease relative to the input concentration. Samples in which cardiac troponin decreased by >60% after Protein G treatment were classified as macrotroponin.

Columns were regenerated by elution with 0.2 M glycine (pH 2.0), followed by washing with TBS, and stored in TBS containing 25% ethanol at 2–8 °C.

To monitor sedimentation and depletion efficiency, Alexa Fluor™ 488–conjugated IgG tracers were added prior to processing and quantified by fluorescence microplate reading. Each centrifugation run included previously characterized macrotroponin negative quality control samples for cardiac troponin I and T, processed in parallel. The mean upper-fraction proportion of cardiac troponin I in the negative macrotroponin I control was 30% (CV 10%), and the corresponding value for cardiac troponin T in the negative macrotroponin T control was 42% (CV 5%). To further assess fractionation performance, ovalbumin labeled with the complementary fluorophore (Alexa Fluor™ 594) was added to the macrotroponin negative control samples. In all cases these internal controls showed excellent performance of the separation of IgG and ovalbumin (data not shown).

Supplemental Tables

Supplemental Table S1. Baseline characteristics of Generation Scotland Scottish Family Health Study (GS:SFHS) and National Health and Nutrition Examination Survey (NHANES).

	**Generation Scotland Scottish Family Health Study (GS:SFHS)** n = 19,499	**National Health and Nutrition Examination Survey (NHANES)** n = 204,513,626
Age (years)	49.0 (36.0 to 58.0)	43.0 (31.0 to 57.0)
Sex (male)	8,125 (41.7%)	97,540,139 (47.7%)
Race/ethnicity		
Black	-	22,440,564 (11.0%)
Hispanic	-	27,125,559 (13.3%)
White	-	145,337,125 (71.1%)
Other	-	9,610,379 (4.7%)
Body mass index (kg/m2)	26.7 (5.2)	28.0 (6.3)
Systolic blood pressure (mmHg)	131.3 (17.8)	122.4 (18.7)
Total cholesterol (mmol/L)	5.1 (1.1)	5.2 (1.1)
High-density lipoprotein cholesterol (mmol/L)	1.5 (0.4)	1.3 (0.4)
Estimated glomerular filtration rate (mL/min/1.73m2)	95.0 (17.4)	102.7 (87.5 to 116.3)
Current smoking (yes)	3,056 (16.2%)	48,102,094 (24.5%)
Family history of cardiovascular disease (yes)	7,403 (38.8%)	28,706,242 (15.1%)
Rheumatoid arthritis (yes)	314 (1.6%)	9,345,915 (5.8%)
Cardiovascular disease at baseline (yes)	877 (4.5%)	13,823,202 (7.0%)
Diabetes mellitus (yes)	562 (2.9%)	13,573,518 (6.6%)
Lipid modifying medication (yes)	1,282 (6.6%)	20,489,900 (10.0%)
Antihypertensive medication (yes)	1,574 (8.1%)	44,768,284 (21.9%)
Cardiac troponin I (ng/L)	1.9 (0.6 to 3.1)	1.7 (0.6 to 2.8)
Cardiac troponin T (ng/L)	3.3 (1.5 to 6.0)	5.0 (3.5 to 7.5)
Cardiac troponin I above limit of blank (yes)	14,578 (74.8%)	145,291,070 (71.0%)
Cardiac troponin T above limit of blank (yes)	10,395 (53.3%)	175,286,283 (85.7%)
Cardiac troponin I above sex-specific 99th percentile (yes)	193 (1.0%)	2,398,662 (1.2%)
Cardiac troponin T above sex-specific 99th percentile (yes)	1,159 (5.9%)	21,617,995 (10.6%)
Cardiac troponin I/T ratio	0.5 (0.4 to 1.1)	0.4 (0.2 to 0.5)

Continuous variables are presented as mean (standard deviation) or median (25th to 75th percentile), as appropriate. Categorical variables are presented as number (%) for the Generation Scotland Scottish Family Health Study (GS:SFHS) and as number of US adults (%) for the National Health and Nutrition Examination Survey (NHANES). Missing values <1% if applicable, except for family history of cardiovascular disease (2.1%), current smoking (3.3%), and SIMD (5.8%) in GS:SFHS and body mass index (2.1%), systolic blood pressure (3.8%), current smoking (4.0%), cardiovascular disease at baseline (4.1%), family history of cardiovascular disease (7.0%), and rheumatoid arthritis (21.2%) in NHANES.

Supplemental Table S2. Suspected macrotroponin complexes and risk of non-fatal myocardial infarction, non-fatal stroke, and cardiovascular death.

Cardiac troponin I above limit of blank	Unadjusted HR (95%CI)	P-value	Adjusted for age and sex HR (95%CI)	P-value	Adjusted for known risk factors* HR (95%CI)	P-value
**Non-fatal myocardial infarction**						
No myocardial injury	reference		reference		reference	
Suspected macrotroponin I	2.76 (1.23 to 6.20)	0.014	2.37 (1.06 to 5.31)	0.036	2.11 (0.94 to 4.75)	0.070
Myocardial injury	3.49 (1.65 to 7.37)	0.001	2.92 (1.38 to 6.20)	0.005	2.55 (1.19 to 5.43)	0.016
**Non-fatal stroke**						
No myocardial injury	reference		reference		reference	
Suspected macrotroponin I	1.40 (0.52 to 3.75)	0.501	1.16 (0.43 to 3.10)	0.768	1.08 (0.40 to 2.90)	0.876
Myocardial injury	4.26 (2.34 to 7.75)	<0.001	2.75 (1.50 to 5.02)	0.001	2.62 (1.43 to 4.81)	0.002
**Cardiovascular death**						
No myocardial injury	reference		reference		reference	
Suspected macrotroponin I	1.85 (0.83 to 4.15)	0.133	1.46 (0.65 to 3.26)	0.359	1.30 (0.58 to 2.92)	0.523
Myocardial injury	7.10 (4.59 to 10.98)	<0.001	3.25 (2.08 to 5.07)	<0.001	3.42 (2.18 to 5.36)	<0.001
**Cardiac troponin T above limit of blank**	**Unadjusted HR (95%CI)**	**P-value**	**Adjusted for age and sex HR (95%CI)**	**P-value**	**Adjusted for known risk factors* HR (95%CI)**	**P-value**
**Non-fatal myocardial infarction**						
No myocardial injury	reference		reference		reference	
Suspected macrotroponin T	0.82 (0.47 to 1.43)	0.484	0.76 (0.43 to 1.34)	0.339	0.77 (0.44 to 1.37)	0.383
Myocardial injury	2.68 (1.81 to 3.96)	<0.001	1.47 (0.96 to 2.24)	0.078	1.44 (0.94 to 2.21)	0.095
**Non-fatal stroke**						
No myocardial injury	reference		reference		reference	
Suspected macrotroponin T	1.56 (1.07 to 2.27)	0.021	1.10 (0.75 to 1.62)	0.632	1.11 (0.75 to 1.63)	0.612
Myocardial injury	4.87 (3.70 to 6.43)	<0.001	2.07 (1.52 to 2.82)	<0.001	1.93 (1.41 to 2.63)	<0.001
**Cardiovascular death**						
No myocardial injury	reference		reference		reference	
Suspected macrotroponin T	2.59 (1.93 to 3.48)	<0.001	1.56 (1.15 to 2.12)	0.005	1.58 (1.16 to 2.15)	0.004
Myocardial injury	9.18 (7.42 to 11.35)	<0.001	2.64 (2.06 to 3.39)	<0.001	2.17 (1.69 to 2.78)	<0.001

The presence of a suspected macrotroponin was defined as a value above the sex-specific 99th percentile upper reference limit of cardiac troponin I or T where significant discordance (more than a three-fold difference) was observed between these assays. Participants without myocardial injury (i.e., those with cardiac troponin values above the limit of blank and within the normal reference range) were used as the referent group. Abbreviations: HR, hazard ratio; CI, confidence interval. * Age, sex, total cholesterol, high-density lipoprotein, systolic blood pressure, smoking status, family history of cardiovascular disease, diabetes, lipid modifying medication, antihypertensive medication, and cardiovascular disease at baseline.

Supplemental Table S3. Suspected macrotroponin complexes and risk of major cardiovascular adverse events, including heart failure hospitalization.

Cardiac troponin I above limit of blank	Unadjusted HR (95%CI)	P-value	Adjusted for age and sex HR (95%CI)	P-value	Adjusted for known risk factors* HR (95%CI)	P-value
No myocardial injury	reference		reference		reference	
Suspected macrotroponin I	1.93 (1.16 to 3.21)	0.011	1.60 (0.96 to 2.65)	0.072	1.50 (0.90 to 2.50)	0.119
Myocardial injury	4.31 (3.00 to 6.19)	<0.001	2.77 (1.92 to 4.00)	<0.001	2.61 (1.81 to 3.77)	<0.001
**Cardiac troponin T above limit of blank**	**Unadjusted HR (95%CI)**	**P-value**	**Adjusted for age and sex HR (95%CI)**	**P-value**	**Adjusted for known risk factors* HR (95%CI)**	**P-value**
No myocardial injury	reference		reference		reference	
Suspected macrotroponin T	1.79 (1.45 to 2.22)	<0.001	1.34 (1.08 to 1.68)	0.009	1.36 (1.09 to 1.69)	0.007
Myocardial injury	5.19 (4.40 to 6.12)	<0.001	2.29 (1.90 to 2.75)	<0.001	2.03 (1.68 to 2.44)	<0.001

The presence of a suspected macrotroponin was defined as a value above the sex-specific 99th percentile upper reference limit of cardiac troponin I or T where significant discordance (more than a three-fold difference) was observed between these assays. Participants without myocardial injury (i.e., those with cardiac troponin values above the limit of blank and within the normal reference range) were used as the referent group. Abbreviations: HR, hazard ratio; CI, confidence interval.

* Age, sex, total cholesterol, high-density lipoprotein, systolic blood pressure, smoking status, family history of cardiovascular disease, diabetes, lipid modifying medication, antihypertensive medication, and cardiovascular disease at baseline.

Supplemental Table S4. Suspected macrotroponin complexes and risk of all-cause death.

Cardiac troponin I above limit of blank	Unadjusted HR (95%CI)	P-value	Adjusted for age and sex HR (95%CI)	P-value	Adjusted for known risk factors* HR (95%CI)	P-value
No myocardial injury	reference		reference		reference	
Suspected macrotroponin I	1.01 (0.48 to 2.12)	0.981	0.81 (0.38 to 1.70)	0.572	0.79 (0.38 to 1.66)	0.535
Myocardial injury	3.93 (2.64 to 5.84)	<0.001	2.04 (1.37 to 3.04)	<0.001	2.18 (1.46 to 3.26)	<0.001
**Cardiac troponin T above limit of blank**	**Unadjusted HR (95%CI)**	**P-value**	**Adjusted for age and sex HR (95%CI)**	**P-value**	**Adjusted for known risk factors* HR (95%CI)**	**P-value**
No myocardial injury	reference		reference		reference	
Suspected macrotroponin T	2.21 (1.80 to 2.72)	<0.001	1.36 (1.10 to 1.69)	0.005	1.36 (1.10 to 1.68)	0.005
Myocardial injury	5.77 (4.89 to 6.81)	<0.001	1.86 (1.54 to 2.24)	<0.001	1.67 (1.38 to 2.01)	<0.001

The presence of a suspected macrotroponin was defined as a value above the sex-specific 99th percentile upper reference limit of cardiac troponin I or T where significant discordance (more than a three-fold difference) was observed between these assays. Participants without myocardial injury (i.e., those with cardiac troponin values above the limit of blank and within the normal reference range) were used as the referent group. Abbreviations: HR, hazard ratio; CI, confidence interval.

* Age, sex, total cholesterol, high-density lipoprotein, systolic blood pressure, smoking status, family history of cardiovascular disease, diabetes, lipid modifying medication, antihypertensive medication, and cardiovascular disease at baseline.

Supplemental Table S5. Suspected macrotroponin complexes (defined as more than a five-fold difference between assays) and risk of major cardiovascular adverse events.

Cardiac troponin I above limit of blank	Unadjusted HR (95%CI)	P-value	Adjusted for age and sex HR (95%CI)	P-value	Adjusted for known risk factors* HR (95%CI)	P-value
No myocardial injury	reference		reference		reference	
Suspected macrotroponin I	1.60 (0.80 to 3.20)	0.188	1.56 (0.78 to 3.13)	0.208	1.41 (0.70 to 2.82)	0.337
Myocardial injury	4.32 (3.09 to 6.05)	<0.001	2.77 (1.97 to 3.89)	<0.001	2.67 (1.90 to 3.75)	<0.001
**Cardiac troponin T above limit of blank**	**Unadjusted HR (95%CI)**	**P-value**	**Adjusted for age and sex HR (95%CI)**	**P-value**	**Adjusted for known risk factors* HR (95%CI)**	**P-value**
No myocardial injury	reference		reference		reference	
Suspected macrotroponin T	1.35 (0.97 to 1.87)	0.073	1.42 (1.02 to 1.97)	0.039	1.42 (1.02 to 1.97)	0.039
Myocardial injury	4.01 (3.42 to 4.70)	<0.001	1.78 (1.49 to 2.13)	<0.001	1.70 (1.42 to 2.04)	<0.001

The presence of a suspected macrotroponin was defined as a value above the sex-specific 99th percentile upper reference limit of cardiac troponin I or T where significant discordance (more than a five-fold difference) was observed between these assays. Participants without myocardial injury (i.e., those with cardiac troponin values above the limit of blank and within the normal reference range) were used as the referent group. Abbreviations: HR, hazard ratio; CI, confidence interval.

* Age, sex, total cholesterol, high-density lipoprotein, systolic blood pressure, smoking status, family history of cardiovascular disease, diabetes, lipid modifying medication, antihypertensive medication, and cardiovascular disease at baseline.

Supplemental Table S6. Suspected macrotroponin complexes and risk of major cardiovascular adverse events in those without cardiovascular disease at baseline.

Cardiac troponin I above limit of blank	Unadjusted HR (95%CI)	P-value	Adjusted for age and sex HR (95%CI)	P-value	Adjusted for known risk factors* HR (95%CI)	P-value
No myocardial injury	reference		reference		reference	
Suspected macrotroponin I	1.54 (0.77 to 3.10)	0.222	1.32 (0.66 to 2.64)	0.438	1.31 (0.65 to 2.64)	0.445
Myocardial injury	4.18 (2.62 to 6.67)	<0.001	2.92 (1.83 to 4.68)	<0.001	2.86 (1.78 to 4.58)	<0.001
**Cardiac troponin T above limit of blank**	**Unadjusted HR (95%CI)**	**P-value**	**Adjusted for age and sex HR (95%CI)**	**P-value**	**Adjusted for known risk factors* HR (95%CI)**	**P-value**
No myocardial injury	reference		reference		reference	
Suspected macrotroponin T	1.83 (1.42 to 2.36)	<0.001	1.34 (1.02 to 1.74)	0.033	1.32 (1.02 to 1.73)	0.038
Myocardial injury	4.36 (3.49 to 5.45)	<0.001	1.92 (1.50 to 2.45)	<0.001	1.91 (1.49 to 2.44)	<0.001

The presence of a suspected macrotroponin was defined as a value above the sex-specific 99th percentile upper reference limit of cardiac troponin I or T where significant discordance (more than a three-fold difference) was observed between these assays. Participants without myocardial injury (i.e., those with cardiac troponin values above the limit of blank and within the normal reference range) were used as the referent group. Abbreviations: HR, hazard ratio; CI, confidence interval.

* Age, sex, total cholesterol, high-density lipoprotein, systolic blood pressure, smoking status, family history of cardiovascular disease, diabetes, lipid modifying medication, antihypertensive medication, and cardiovascular disease at baseline.

Supplemental Table S7. Suspected macrotroponin complexes and risk of major cardiovascular adverse events in those with cardiac troponin concentrations above the limit of detection.

Cardiac troponin I above limit of detection	Unadjusted HR (95%CI)	P-value	Adjusted for age and sex HR (95%CI)	P-value	Adjusted for known risk factors* HR (95%CI)	P-value
No myocardial injury	reference		reference		reference	
Suspected macrotroponin I	1.58 (0.93 to 2.68)	0.089	1.62 (0.95 to 2.74)	0.074	1.53 (0.90 to 2.60)	0.115
Myocardial injury	3.68 (2.54 to 5.32)	<0.001	2.96 (2.04 to 4.30)	<0.001	2.84 (1.95 to 4.13)	<0.001
**Cardiac troponin T above limit of detection**	**Unadjusted HR (95%CI)**	**P-value**	**Adjusted for age and sex HR (95%CI)**	**P-value**	**Adjusted for known risk factors* HR (95%CI)**	**P-value**
No myocardial injury	reference		reference		reference	
Suspected macrotroponin T	1.28 (1.01 to 1.62)	0.038	1.15 (0.90 to 1.47)	0.261	1.16 (0.91 to 1.49)	0.233
Myocardial injury	3.94 (3.29 to 4.71)	<0.001	2.04 (1.67 to 2.51)	<0.001	1.87 (1.52 to 2.29)	<0.001

The presence of a suspected macrotroponin was defined as a value above the sex-specific 99th percentile upper reference limit of cardiac troponin I or T where significant discordance (more than a three-fold difference) was observed between these assays. Participants without myocardial injury (i.e., those with cardiac troponin values above the limit of detection and within the normal reference range) were used as the referent group. Abbreviations: HR, hazard ratio; CI, confidence interval.

* Age, sex, total cholesterol, high-density lipoprotein, systolic blood pressure, smoking status, family history of cardiovascular disease, diabetes, lipid modifying medication, antihypertensive medication, and cardiovascular disease at baseline.

Supplemental Table S8. Baseline characteristics without survey weights of participants with cardiac troponin I concentrations above the limit of blank and/or assay discordance in the National Health and Nutrition Examination Survey (NHANES).

	Cardiac troponin I ≤sex-specific URL	Cardiac troponin I >sex-specific URL	
	**No myocardial injury** n = 9,512	**Suspected macrotroponin I*** n = 27	**Myocardial injury** n = 198
Age (years)	53.0 (36.0 to 69.0)	42.0 (34.0 to 62.0)	75.0 (64.0 to 83.0)
Sex (male)	5,466 (57.5%)	18 (66.7%)	63 (31.8%)
Race/ethnicity			
Black	1,823 (19.2%)	7 (25.9%)	66 (33.3%)
Hispanic	2,469 (26.0%)	9 (33.3%)	30 (15.2%)
White	327 (3.4%)	1 (3.7%)	7 (3.5%)
Other	4,893 (51.4%)	10 (37.0%)	95 (48.0%)
Body mass index (kg/m2)	28.6 (6.2)	26.6 (5.0)	28.3 (6.4)
Systolic blood pressure (mmHg)	128.9 (21.1)	128.6 (21.8)	142.3 (28.1)
Total cholesterol (mmol/L)	5.3 (1.1)	4.8 (0.9)	5.4 (1.2)
High-density lipoprotein cholesterol (mmol/L)	1.3 (0.4)	1.4 (0.3)	1.4 (0.5)
eGFR (mL/min/1.73m2)	96.5 (79.8, 111.4)	102.8 (87.6, 114.2)	56.6 (41.6, 79.5)
Current smoking (yes)	1,875 (21.1%)	7 (26.9%)	25 (12.8%)
Family history of cardiovascular disease (yes)	1,064 (12.4%)	2 (8.0%)	17 (9.4%)
Rheumatoid arthritis (yes)	606 (8.9%)	0 (0.0%)	24 (19.4%)
Cardiovascular disease at baseline (yes)	997 (11.2%)	3 (11.5%)	71 (37.4%)
Diabetes mellitus (yes)	1,026 (10.8%)	4 (14.8%)	48 (24.2%)
Lipid modifying medication (yes)	1,266 (13.3%)	5 (18.5%)	50 (25.3%)
Antihypertensive medication (yes)	3,023 (31.8%)	7 (25.9%)	152 (76.8%)
Cardiac troponin I (ng/L)	2.4 (1.7 to 3.9)	43.4 (26.1 to 67.2)	37.8 (19.9 to 58.0)
Cardiac troponin T (ng/L)	6.4 (4.5 to 10.4)	8.3 (5.1 to 11.7)	31.9 (20.3 to 52.6)
Cardiac troponin T above limit of blank (yes)	8,865 (93.2%)	25 (92.6%)	198 (100.0%)
Cardiac troponin T above sex-specific 99th percentile (yes)	1,756 (18.5%)	0 (0.0%)	187 (94.4%)
Cardiac troponin I/T ratio	0.4 (0.3 to 0.6)	5.4 (3.7 to 10.0)	1.2 (0.8 to 2.0)

Continuous variables are presented as mean (standard deviation) or median (25th to 75th percentile), as appropriate. Categorical variables are presented as number (%).
*Suspected macrotroponin is defined as a value above the sex-specific 99th percentile of cardiac troponin I or T where significant discordance (more than a three-fold difference) was observed between these assays.

Supplemental Table S9. Baseline characteristics without survey weights of participants with cardiac troponin T concentrations above the limit of blank and/or assay discordance in the National Health and Nutrition Examination Survey (NHANES).

	Cardiac troponin T ≤sex-specific URL	Cardiac troponin T >sex-specific URL	
	**No myocardial injury** n = 9,802	**Suspected macrotroponin T*** n = 1,089	**Myocardial injury** n = 893
Age (years)	43.0 (28.0 to 60.0)	75.0 (66.0 to 82.0)	76.0 (67.0 to 83.0)
Sex (male)	5,462 (55.7%)	493 (45.3%)	375 (42.0%)
Race/ethnicity			
Black	1,847 (18.8%)	166 (15.2%)	187 (20.9%)
Hispanic	2,946 (30.1%)	206 (18.9%)	148 (16.6%)
White	373 (3.8%)	20 (1.8%)	25 (2.8%)
Other	4,636 (47.3%)	697 (64.0%)	533 (59.7%)
Body mass index (kg/m2)	28.0 (6.2)	28.4 (6.1)	28.8 (6.5)
Systolic blood pressure (mmHg)	123.4 (18.8)	137.7 (23.4)	145.3 (26.7)
Total cholesterol (mmol/L)	5.2 (1.1)	5.3 (1.2)	5.3 (1.2)
High-density lipoprotein cholesterol (mmol/L)	1.3 (0.4)	1.4 (0.4)	1.4 (0.4)
eGFR (mL/min/1.73m2)	102.8 (89.3, 117.1)	72.2 (55.2, 89.6)	67.4 (51.4, 86.3)
Current smoking (yes)	2,036 (23.4%)	144 (13.3%)	105 (11.8%)
Family history of cardiovascular disease (yes)	1,066 (12.6%)	95 (9.1%)	103 (12.3%)
Rheumatoid arthritis (yes)	470 (6.5%)	115 (18.5%)	106 (19.6%)
Cardiovascular disease at baseline (yes)	557 (6.4%)	242 (22.5%)	309 (35.3%)
Diabetes mellitus (yes)	673 (6.9%)	289 (26.5%)	210 (23.6%)
Lipid modifying medication (yes)	906 (9.2%)	246 (22.6%)	244 (27.3%)
Antihypertensive medication (yes)	2,004 (20.4%)	666 (61.2%)	631 (70.7%)
Cardiac troponin I (ng/L)	1.7 (0.6 to 2.7)	3.5 (2.4 to 5.2)	10.6 (6.9 to 19.0)
Cardiac troponin T (ng/L)	5.3 (4.0 to 7.2)	18.6 (13.2 to 27.3)	18.8 (12.9 to 26.8)
Cardiac troponin I above limit of blank (yes)	7,145 (72.9%)	1,050 (96.4%)	893 (100.0%)
Cardiac troponin I above sex-specific 99th percentile (yes)	36 (0.4%)	0 (0.0%)	187 (20.9%)
Cardiac troponin I/T ratio	0.3 (0.2 to 0.5)	0.2 (0.1 to 0.3)	0.5 (0.4 to 0.8)

Continuous variables are presented as mean (standard deviation) or median (25th to 75th percentile), as appropriate. Categorical variables are presented as number (%).
*Suspected macrotroponin is defined as a value above the sex-specific 99th percentile of cardiac troponin I or T where significant discordance (more than a three-fold difference) was observed between these assays.

Supplemental Table S10. Baseline characteristics of participants with cardiac troponin I concentrations above the limit of blank and/or assay discordance in the National Health and Nutrition Examination Survey (NHANES)

	Cardiac troponin I ≤sex-specific URL	Cardiac troponin I >sex-specific URL	
	**No myocardial injury** n = 142,892,408	**Suspected macrotroponin I*** n = 323,093	**Myocardial injury** n = 2,075,567
Age (years)	48.0 (36.0 to 62.0)	40.0 (29.0 to 52.0)	71.0 (59.0 to 80.0)
Sex (male)	81,338,640 (56.9%)	241,047 (74.6%)	588,333 (28.3%)
Race/ethnicity			
Black	15,727,420 (11.0%)	64,884 (20.1%)	534,446 (25.7%)
Hispanic	17,057,593 (11.9%)	39,298 (12.2%)	101,895 (4.9%)
White	103,816,439 (72.7%)	205,833 (63.7%)	1,317,241 (63.5%)
Other	6,290,957 (4.4%)	13,079 (4.0%)	121,986 (5.9%)
Body mass index (kg/m2)	28.7 (6.3)	26.0 (4.7)	28.4 (6.5)
Systolic blood pressure (mmHg)	126.2 (19.1)	122.0 (15.8)	139.0 (27.8)
Total cholesterol (mmol/L)	5.3 (1.1)	4.9 (1.0)	5.4 (1.2)
High-density lipoprotein cholesterol (mmol/L)	1.3 (0.4)	1.4 (0.3)	1.4 (0.5)
eGFR (mL/min/1.73m2)	98.8 (83.7, 112.6)	97.0 (87.6, 113.3)	63.3 (42.9, 86.3)
Current smoking (yes)	32,762,674 (23.5%)	83,237 (25.9%)	356,457 (17.3%)
Family history of cardiovascular disease (yes)	20,026,699 (14.9%)	50,728 (15.9%)	269,525 (14.3%)
Rheumatoid arthritis (yes)	7,522,091 (6.8%)	0 (0.0%)	215,083 (16.8%)
Cardiovascular disease at baseline (yes)	11,928,950 (8.6%)	40,043 (12.4%)	845,140 (41.9%)
Diabetes mellitus (yes)	11,431,109 (8.0%)	38,934 (12.1%)	499,718 (24.1%)
Lipid modifying medication (yes)	18,091,408 (12.7%)	48,695 (15.1%)	708,176 (34.1%)
Antihypertensive medication (yes)	39,302,900 (27.5%)	67,071 (20.8%)	1,580,263 (76.1%)
Cardiac troponin I (ng/L)	2.2 (1.6 to 3.3)	48.4 (34.1 to 61.7)	36.9 (19.7 to 57.1)
Cardiac troponin T (ng/L)	5.9 (4.2 to 8.6)	8.3 (5.1 to 10.7)	29.8 (18.6 to 49.0)
Cardiac troponin T above limit of blank (yes)	131,028,187 (91.7%)	294,482 (91.1%)	2,075,568 (100.0%)
Cardiac troponin T above sex-specific 99th percentile (yes)	19,108,518 (13.4%)	0 (0.0%)	1,961,250 (94.5%)
Cardiac troponin I/T ratio	0.4 (0.3 to 0.6)	6.8 (3.7 to 10.0)	1.2 (0.8 to 2.2)

Continuous variables are presented as mean (standard deviation) or median (25th to 75th percentile), as appropriate. Categorical variables are presented as number of US adults (%). *Suspected macrotroponin is defined as a value above the sex-specific 99th percentile upper reference limit of cardiac troponin I or T where significant discordance (more than a three-fold difference) was observed between these assays.

Supplemental Table S11. Baseline characteristics of participants with cardiac troponin T concentrations above the limit of blank and/or assay discordance in the National Health and Nutrition Examination Survey (NHANES).

	Cardiac troponin T ≤sex-specific URL	Cardiac troponin T >sex-specific URL	
	**No myocardial injury** n = 153,668,288	**Suspected macrotroponin T*** n = 12,144,669	**Myocardial injury** n = 9,473,324
Age (years)	43.0 (31.0 to 55.0)	72.0 (59.0 to 80.0)	74.0 (64.0 to 81.0)
Sex (male)	85,340,378 (55.5%)	4,838,251 (39.8%)	3,288,964 (34.7%)
Race/ethnicity			
Black	16,187,183 (10.5%)	1,219,060 (10.0%)	1,425,956 (15.1%)
Hispanic	21,201,129 (13.8%)	804,294 (6.6%)	590,189 (6.2%)
White	108,665,523 (70.7%)	9,831,794 (81.0%)	7,136,595 (75.3%)
Other	7,614,454 (5.0%)	289,522 (2.4%)	320,585 (3.4%)
Body mass index (kg/m2)	28.1 (6.2)	28.7 (6.4)	29.0 (6.6)
Systolic blood pressure (mmHg)	122.2 (17.3)	134.8 (22.8)	142.6 (25.7)
Total cholesterol (mmol/L)	5.2 (1.1)	5.3 (1.2)	5.4 (1.2)
High-density lipoprotein cholesterol (mmol/L)	1.3 (0.4)	1.4 (0.5)	1.4 (0.4)
eGFR (mL/min/1.73m2)	102.6 (89.2, 114.7)	74.0 (57.5, 91.4)	70.4 (52.4, 89.0)
Current smoking (yes)	37,375,871 (25.4%)	1,715,252 (14.2%)	1,193,795 (12.6%)
Family history of cardiovascular disease (yes)	21,401,596 (15.0%)	1,343,785 (11.5%)	1,392,653 (15.7%)
Rheumatoid arthritis (yes)	6,514,462 (5.3%)	1,073,893 (16.1%)	1,095,241 (19.6%)
Cardiovascular disease at baseline (yes)	7,730,331 (5.3%)	2,423,633 (20.1%)	3,320,818 (35.6%)
Diabetes mellitus (yes)	8,286,077 (5.4%)	2,845,177 (23.4%)	2,070,800 (21.9%)
Lipid modifying medication (yes)	14,258,567 (9.3%)	2,919,872 (24.0%)	3,012,975 (31.8%)
Antihypertensive medication (yes)	29,209,624 (19.0%)	7,078,716 (58.3%)	6,563,183 (69.3%)
Cardiac troponin I (ng/L)	1.7 (0.6 to 2.6)	3.1 (2.2 to 4.8)	9.9 (6.4 to 17.8)
Cardiac troponin T (ng/L)	5.1 (4.0 to 6.9)	17.7 (12.5 to 24.0)	17.2 (12.2 to 24.0)
Cardiac troponin I above limit of blank (yes)	112,328,470 (73.1%)	11,596,442 (95.5%)	9,473,325 (100.0%)
Cardiac troponin I above sex-specific 99th percentile (yes)	408,801 (0.3%)	0 (0.0%)	1,961,250 (20.7%)
Cardiac troponin I/T ratio	0.3 (0.2 to 0.5)	0.2 (0.1 to 0.3)	0.5 (0.4 to 0.8)

Continuous variables are presented as mean (standard deviation) or median (25th to 75th percentile), as appropriate. Categorical variables are presented as number of US adults (%). *Suspected macrotroponin is defined as a value above the sex-specific 99th percentile upper reference limit of cardiac troponin I or T where significant discordance (more than a three-fold difference) was observed between these assays.

Supplemental Table S12. Suspected macrotroponin complexes and risk of cardiovascular death in the National Health and Nutrition Examination Survey.

Cardiac troponin I above limit of blank	Unadjusted HR (95%CI)	P-value	Adjusted for age and sex HR (95%CI)	P-value	Adjusted for known risk factors* HR (95%CI)	P-value
No myocardial injury	reference		reference		reference	
Suspected macrotroponin I	1.15 (0.25 to 5.25)	0.860	1.85 (0.39 to 8.72)	0.438	0.67 (0.08 to 5.31)	0.706
Myocardial injury	9.36 (6.77 to 12.93)	<0.001	3.38 (2.47 to 4.63)	<0.001	2.14 (1.38 to 3.33)	0.001
**Cardiac troponin T above limit of blank**	**Unadjusted HR (95%CI)**	**P-value**	**Adjusted for age and sex HR (95%CI)**	**P-value**	**Adjusted for known risk factors* HR (95%CI)**	**P-value**
No myocardial injury	reference		reference		reference	
Suspected macrotroponin T	8.69 (7.00 to 10.77)	<0.001	2.19 (1.74 to 2.75)	<0.001	1.80 (1.44 to 2.25)	<0.001
Myocardial injury	16.38 (13.26 to 20.24)	<0.001	3.74 (3.06 to 4.56)	<0.001	2.65 (2.14 to 3.28)	<0.001

The presence of a suspected macrotroponin was defined as a value above the sex-specific 99th percentile upper reference limit of cardiac troponin I or T where significant discordance (more than a three-fold difference) was observed between these assays. Participants without myocardial injury (i.e., those with cardiac troponin values above the limit of blank and within the normal reference range) were used as the referent group. Abbreviations: HR, hazard ratio; CI, confidence interval.

* Age, sex, total cholesterol, high-density lipoprotein, systolic blood pressure, smoking status, family history of cardiovascular disease, diabetes, lipid modifying medication, antihypertensive medication, and cardiovascular disease at baseline.

Supplemental Figures

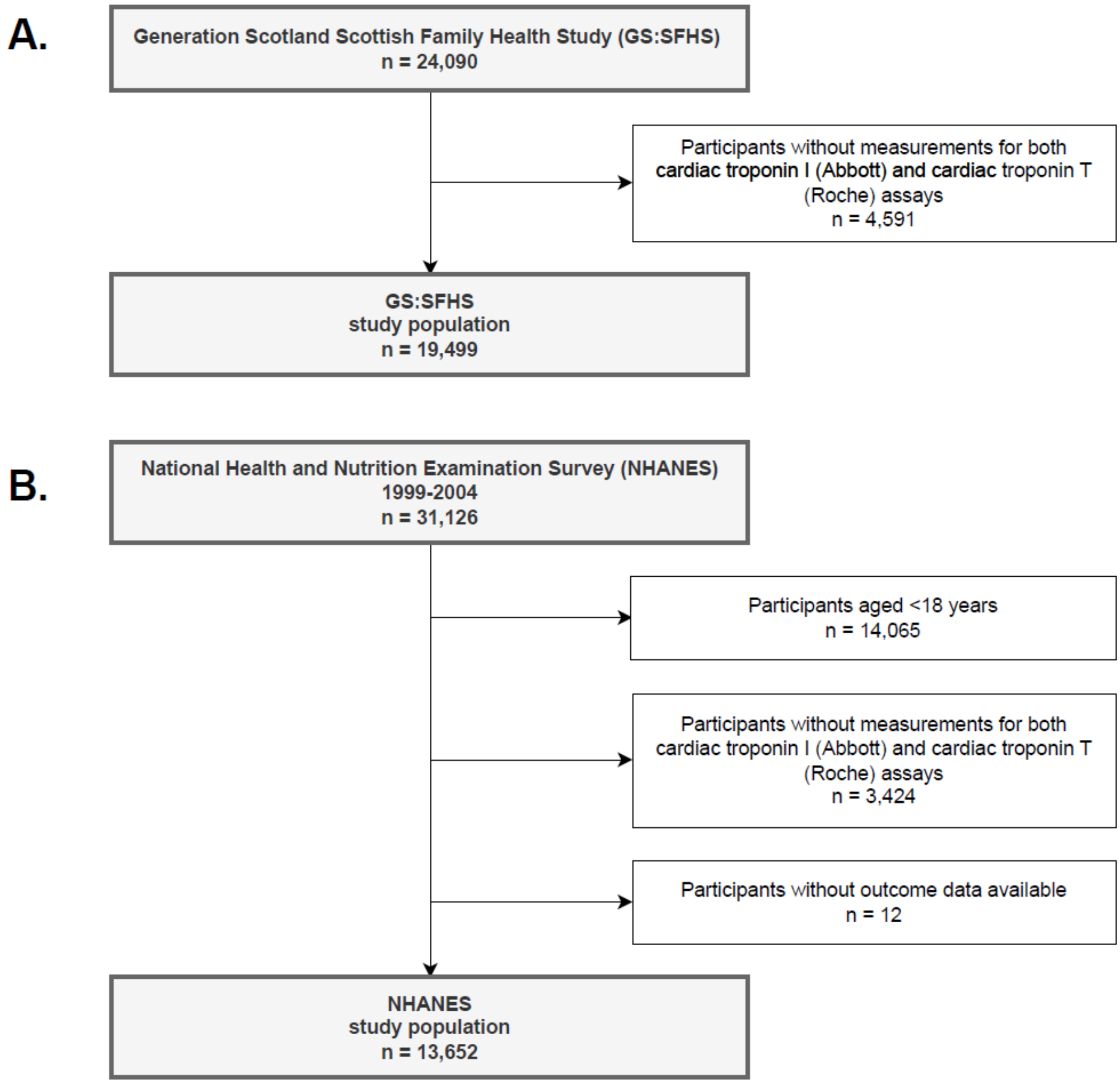

Supplemental Figure S1. Flowchart of study populations. (A) Generation Scotland Scottish Family Health Study (GS:SFHS). (B) National Health and Nutrition Examination Survey (NHANES).

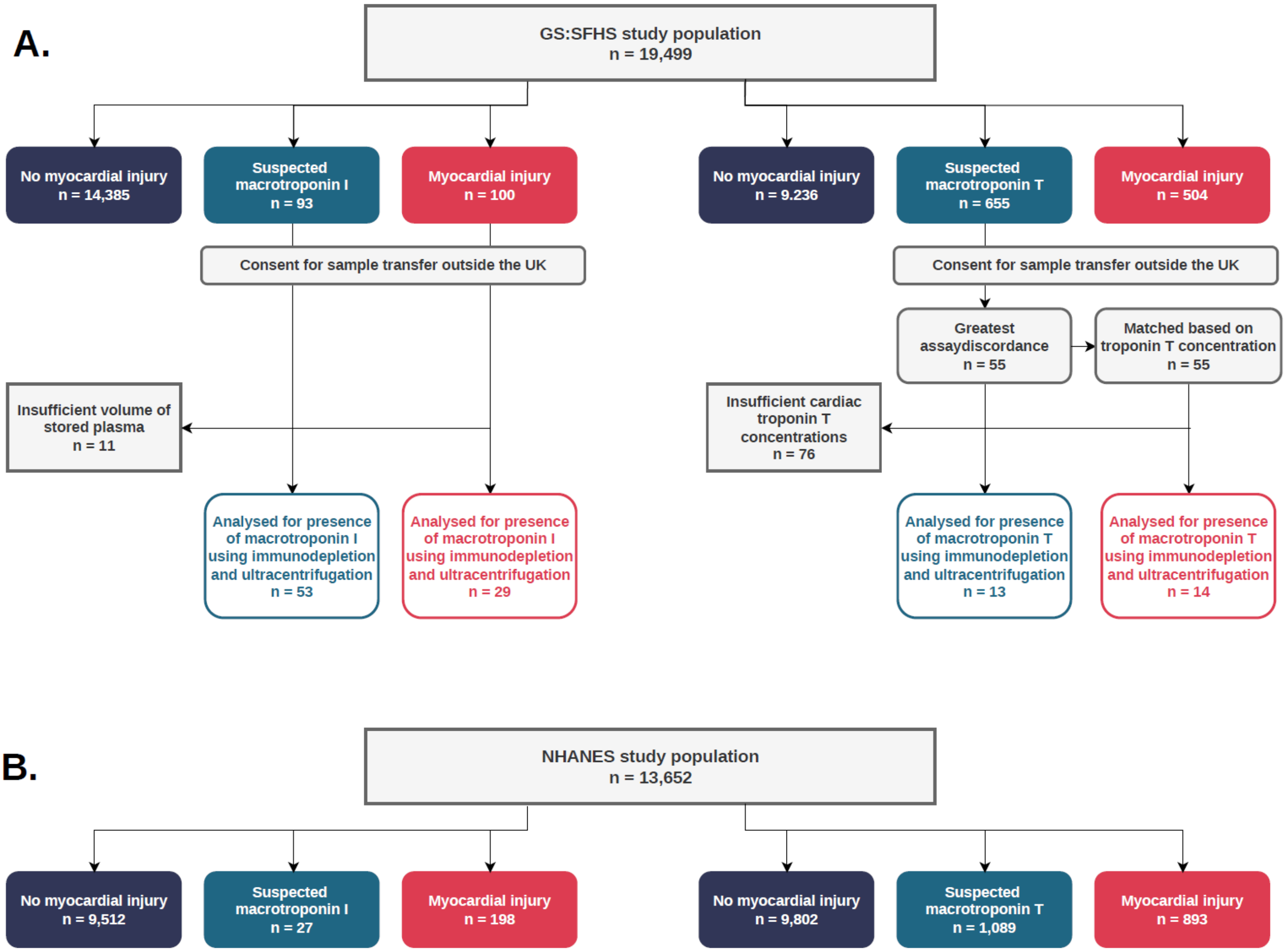

Supplemental Figure S2. Flowchart of macrotroponin grouping and biochemical analysis in (A) the Generation Scotland Scottish Family Health Study (GS:SFHS) and (B) National Health and Nutrition Examination Survey (NHANES). The presence of a suspected macrotroponin was defined a priori as a value above the sex-specific 99th percentile URL of cardiac troponin I or T where significant discordance (more than a three-fold difference in absolute concentration) was observed between these assays. Participants were categorized into three groups: (1) No myocardial injury: those with cardiac troponin values above the limit of blank but within the normal reference range, (2) Suspected macrotroponin: those with cardiac troponin concentrations above the sex-specific 99th

percentile URL with assay discordance, and (3) Myocardial injury: those with cardiac troponin concentrations above the sex-specific 99th percentile URL without assay discordance. In addition, individuals with both cardiac troponin I and cardiac troponin T concentrations above their respective sex-specific 99th percentile URLs were classified as myocardial injury. To investigate the mechanisms of assay discordance and the proportion attributable to macrotroponin complexes, two complementary methods recommended by the International Federation of Clinical Chemistry (IFCC) were applied to a subset of samples from GS:SFHS cohort study. Only samples from participants who had provided consent for their transfer outside the United Kingdom were included. All available samples from individuals with cardiac troponin I concentrations above the 99th percentile URL, both with and without assay discordance were evaluated. Among individuals with cardiac troponin T above the 99th percentile URL, the 55 participants with the greatest assay discordance were selected and matched based on cardiac troponin T concentration (±5 ng/L) to 55 participants with elevated cardiac troponin T but no discordance. Further analysis to confirm the presence of macrotroponin complexes in NHANES samples with discordant cardiac troponin concentrations was not undertaken.

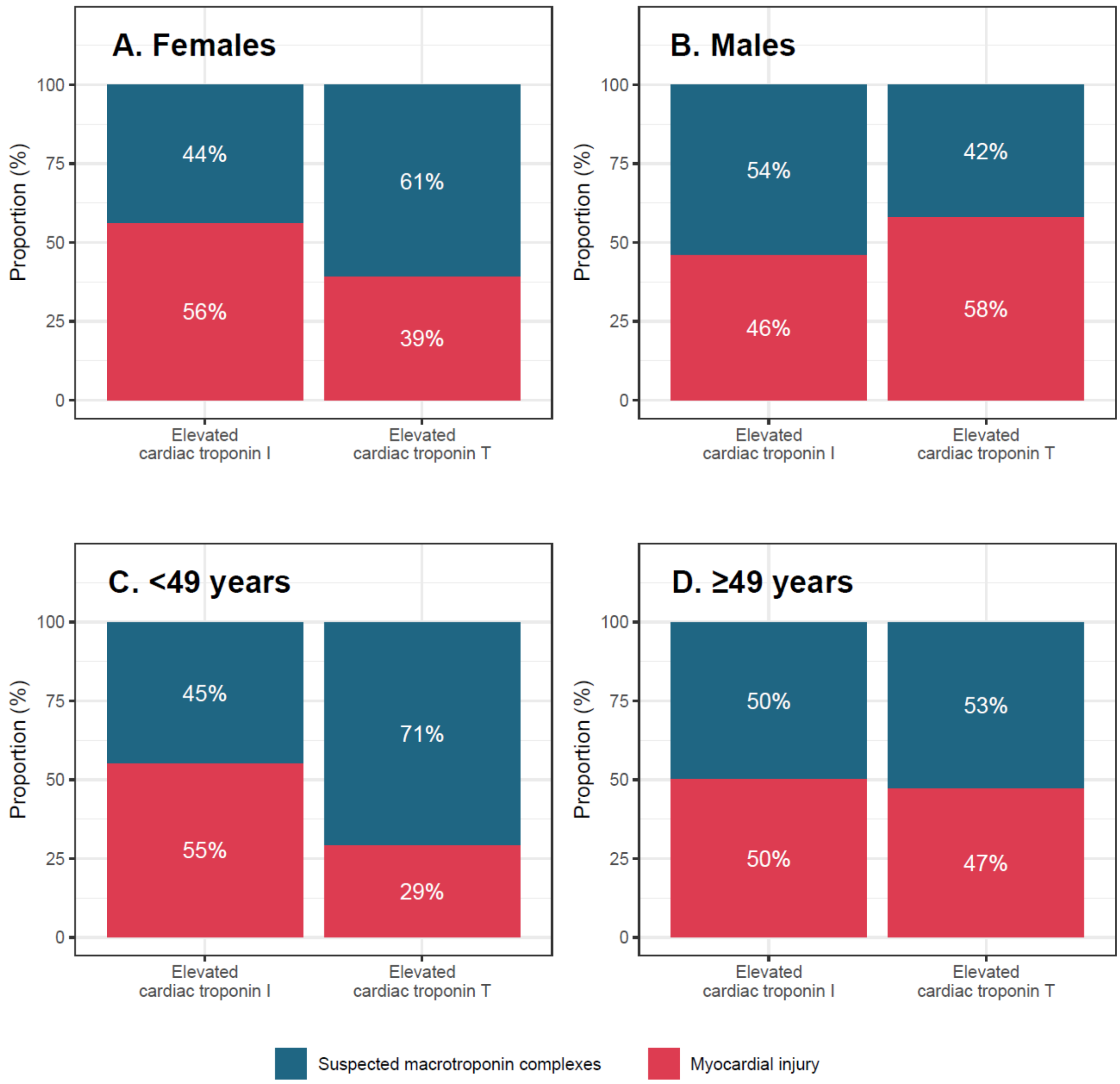

Supplemental Figure S3. Prevalence of suspected macrotroponin complexes stratified by sex and median age in GS:SFHS. The proportion of individuals with suspected macrotroponin complexes or myocardial injury among those with elevated cardiac troponin levels, stratified by sex (females [A] and males [B]) and by median age (<49 years [C] and ≥49 years [D]). Suspected macrotroponin was defined as a value above the sex-specific 99th percentile upper reference limit of cardiac troponin I or T where significant discordance (more than a three-fold difference) was observed between these assays.

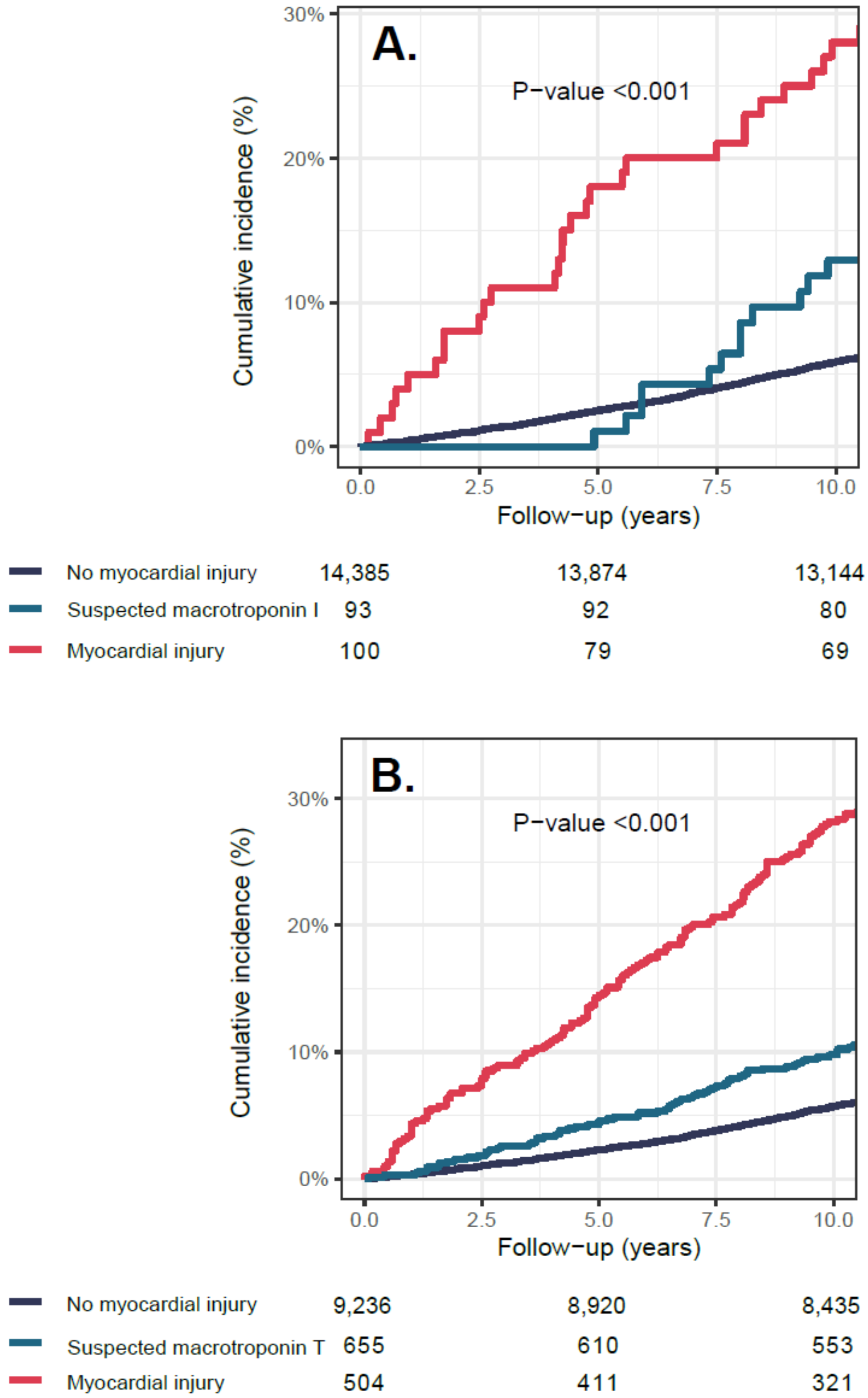

Supplemental Figure S4. Cumulative incidence of major adverse cardiovascular events during follow-up in participants with and without suspected macrotroponin I (A) and T (B) complexes. The presence of a suspected macrotroponin was defined as a value above the sex-specific 99th percentile upper reference limit of cardiac troponin I or T where significant discordance (more than a three-fold difference in absolute concentration) was observed between these assays. Major adverse cardiovascular events included non-fatal myocardial infarction, non-fatal ischemic stroke or transient ischemic attack, or cardiovascular death.

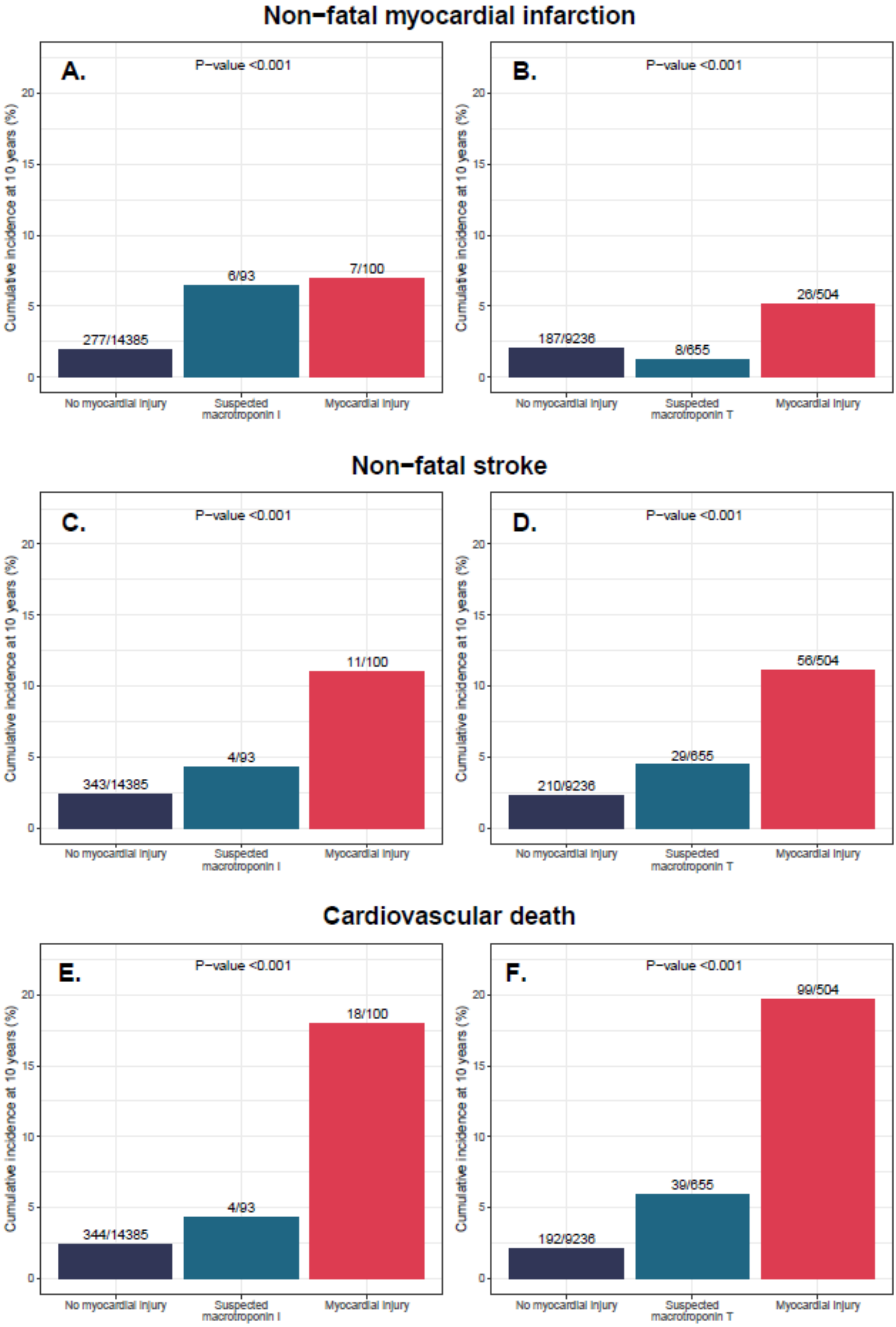

Supplemental Figure S5. Cumulative incidence of non-fatal myocardial infarction, non-fatal stroke, and cardiovascular death at 10 years in participants with and without suspected macrotroponin I (A, C, and E) and T (B, D, and F) complexes. The presence of a suspected macrotroponin was defined as a value above the sex-specific 99th percentile upper reference limit of cardiac troponin I or T where significant discordance (more than a three-fold difference) was observed between these assays.

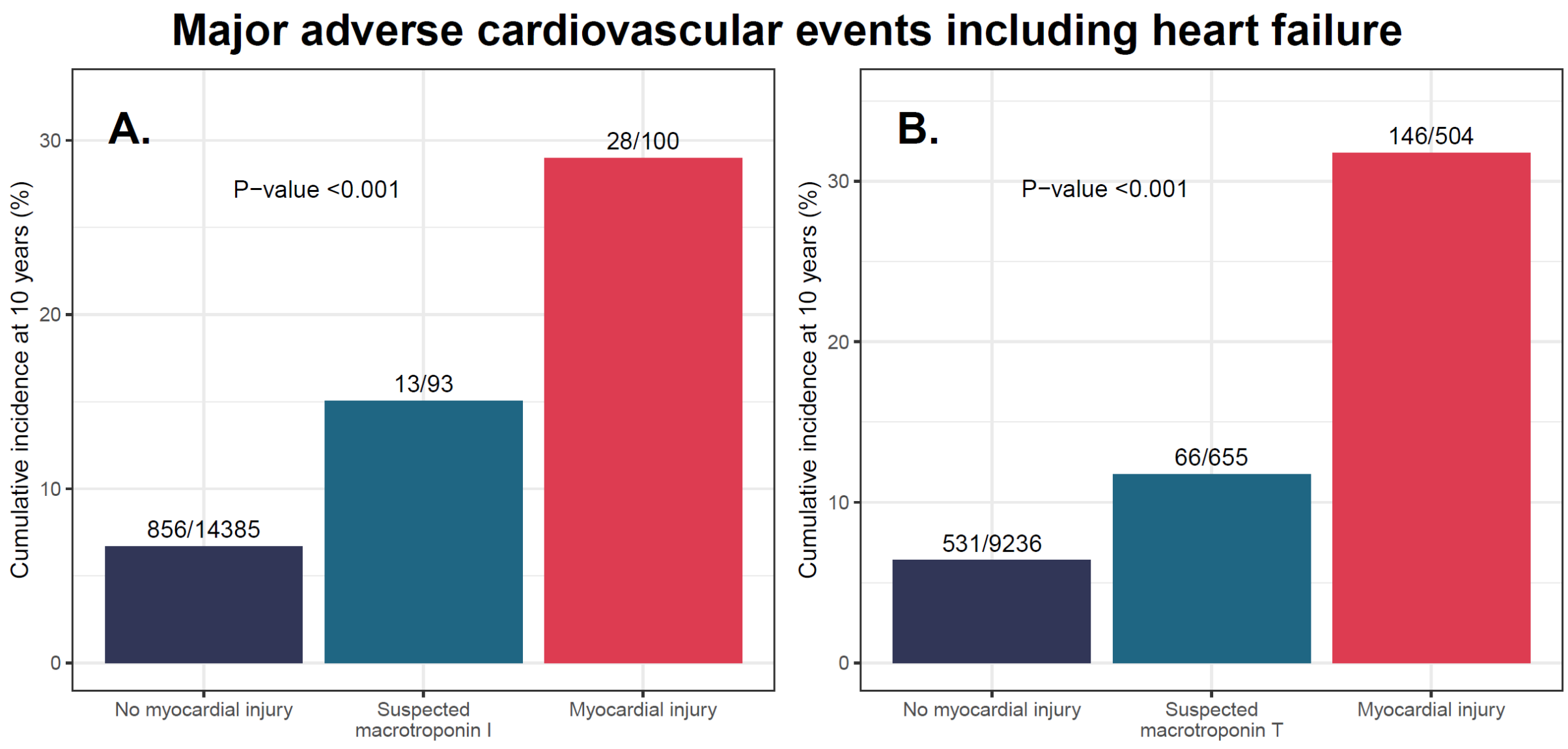

Supplemental Figure S6. Cumulative incidence of major adverse cardiovascular evens including heart failure at 10 years in participants with and without suspected macrotroponin I (A) and T (B) complexes. The presence of a suspected macrotroponin was defined as a value above the sex-specific 99th percentile upper reference limit of cardiac troponin I or T where significant discordance (more than a three-fold difference) was observed between these assays.

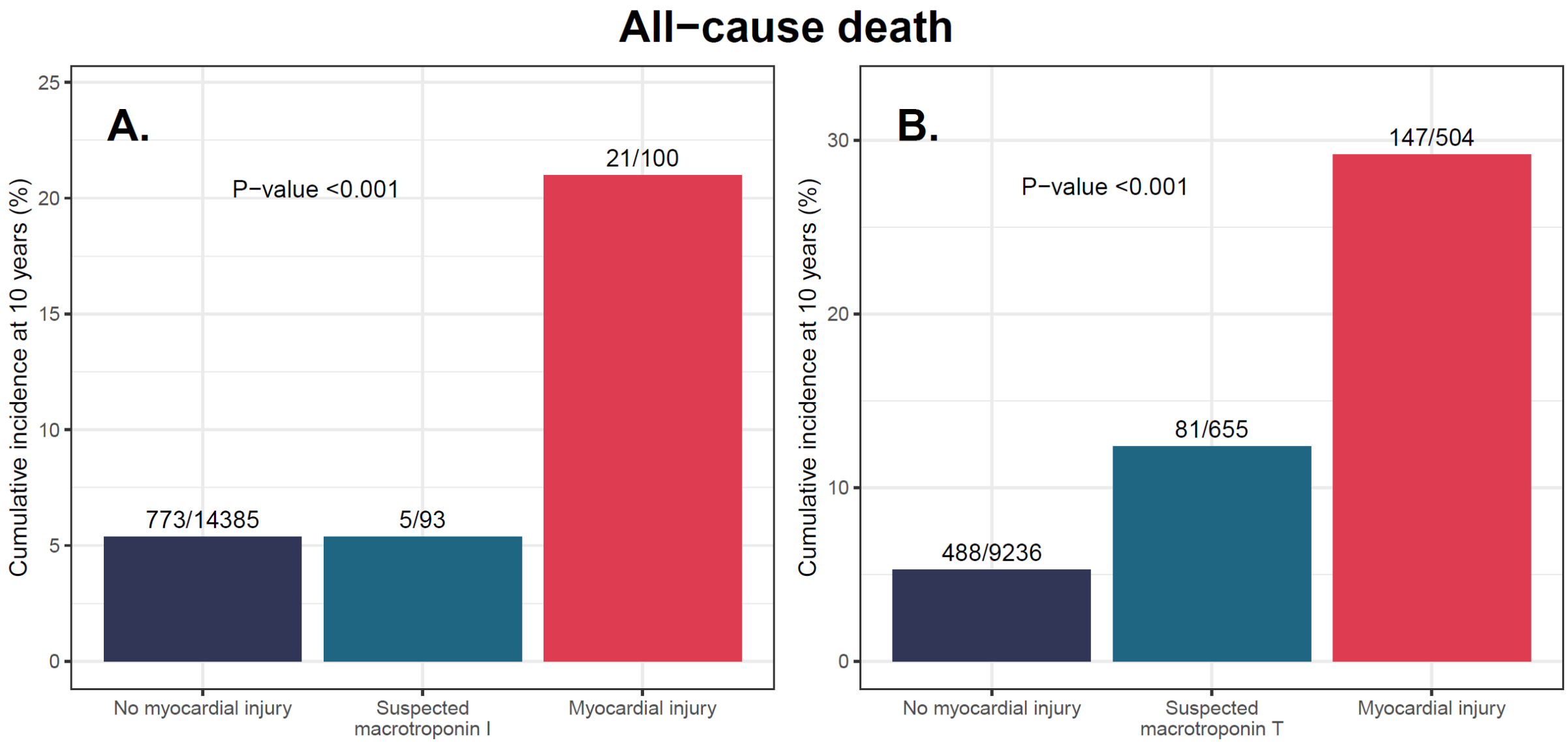

Supplemental Figure S7. Cumulative incidence of all-cause death at 10 years in participants with and without suspected macrotroponin I (A) and T (B) complexes. The presence of a suspected macrotroponin was defined as a value above the sex-specific 99th percentile upper reference limit of cardiac troponin I or T where significant discordance (more than a three-fold difference) was observed between these assays.

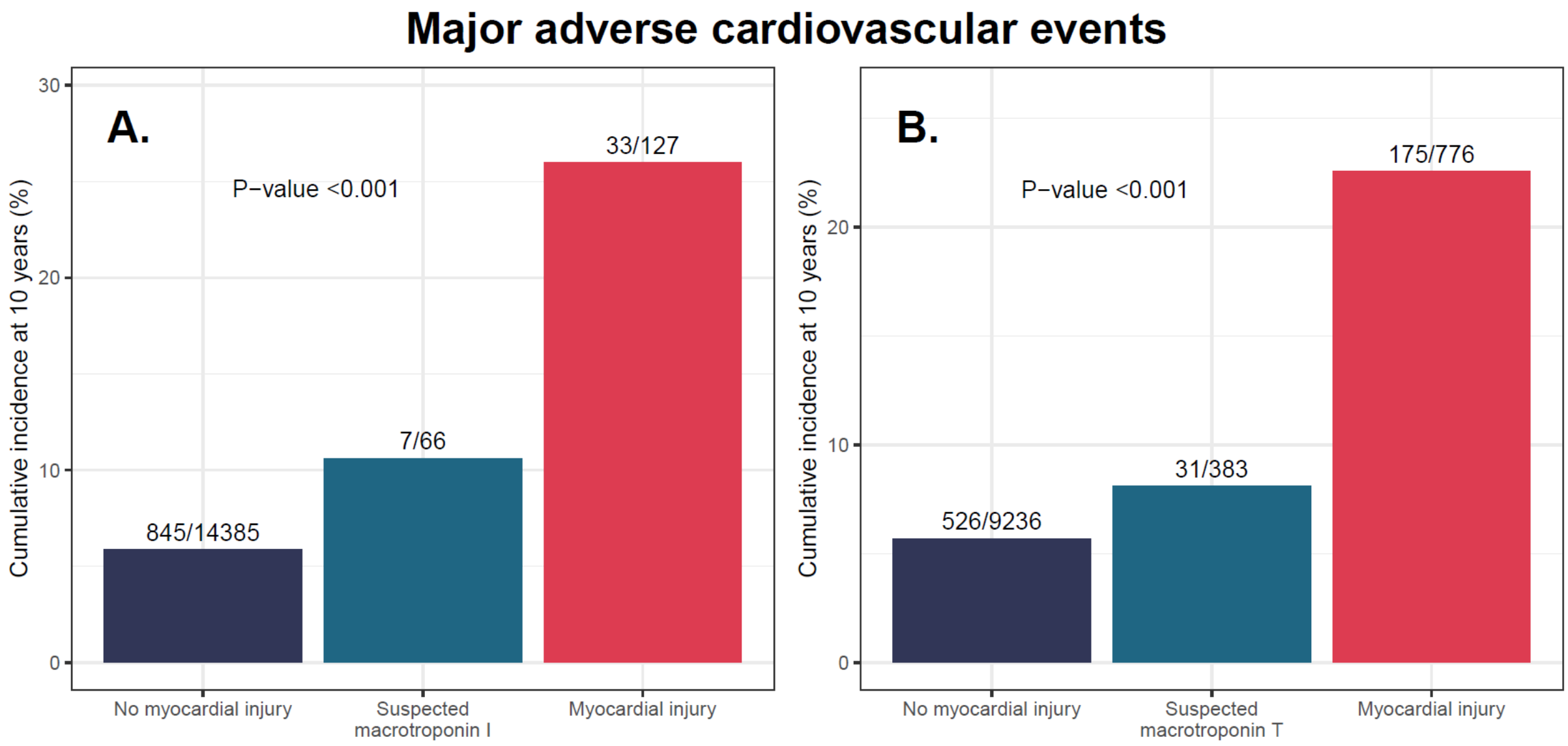

Supplemental Figure S8. Cumulative incidence of major adverse cardiovascular events at 10 years in participants with and without suspected macrotroponin I (A) and T (B) complexes (defined as more than a five-fold difference between assays). The presence of a suspected macrotroponin was defined as a value above the sex-specific 99th percentile upper reference limit of cardiac troponin I or T where significant discordance (more than a five-fold difference) was observed between these assays.

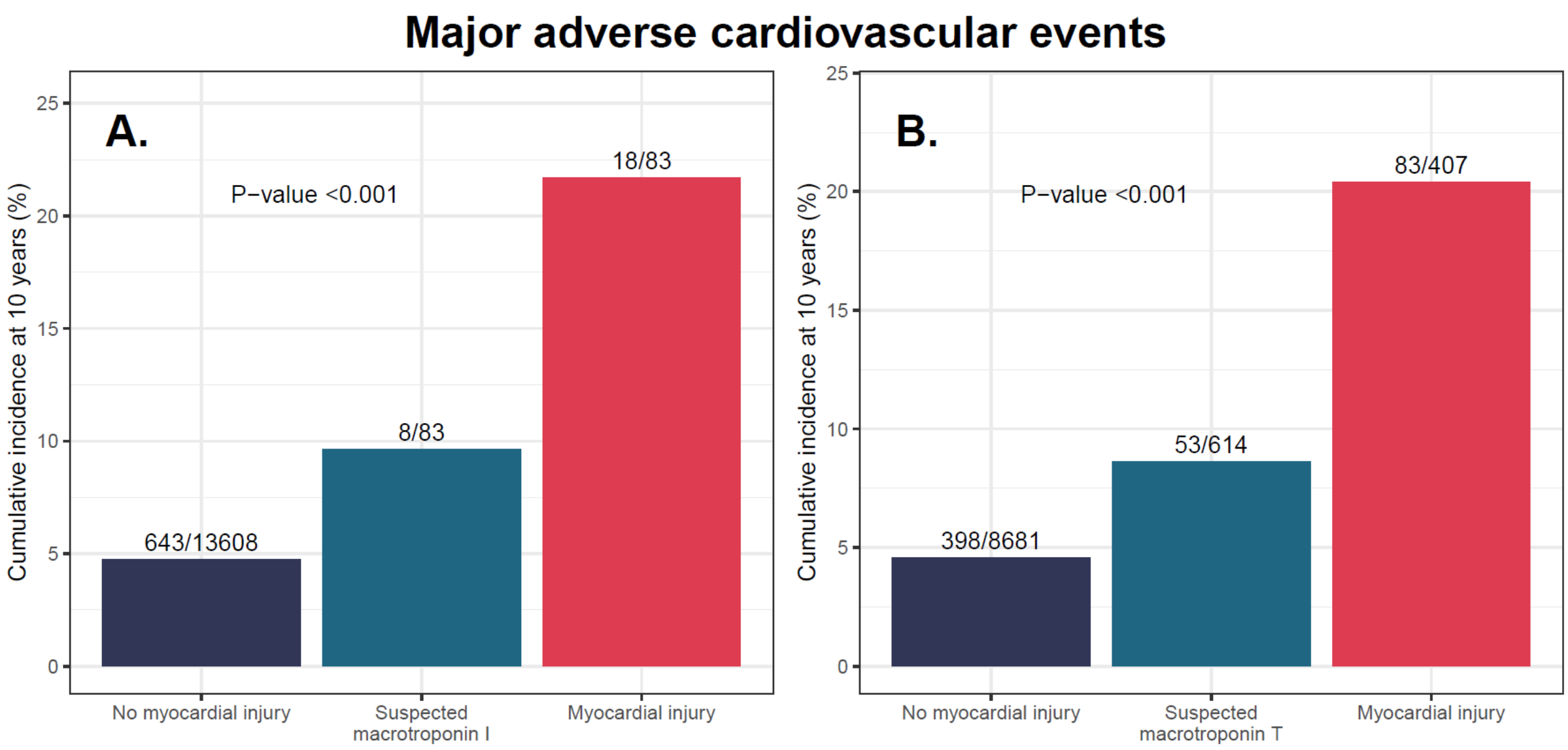

Supplemental Figure S9. Cumulative incidence of major adverse cardiovascular events at 10 years in participants with and without suspected macrotroponin I (A) and T (B) complexes in those without cardiovascular disease at baseline. The presence of a suspected macrotroponin was defined as a value above the sex-specific 99th percentile upper reference limit of cardiac troponin I or T where significant discordance (more than a three-fold difference) was observed between these assays.

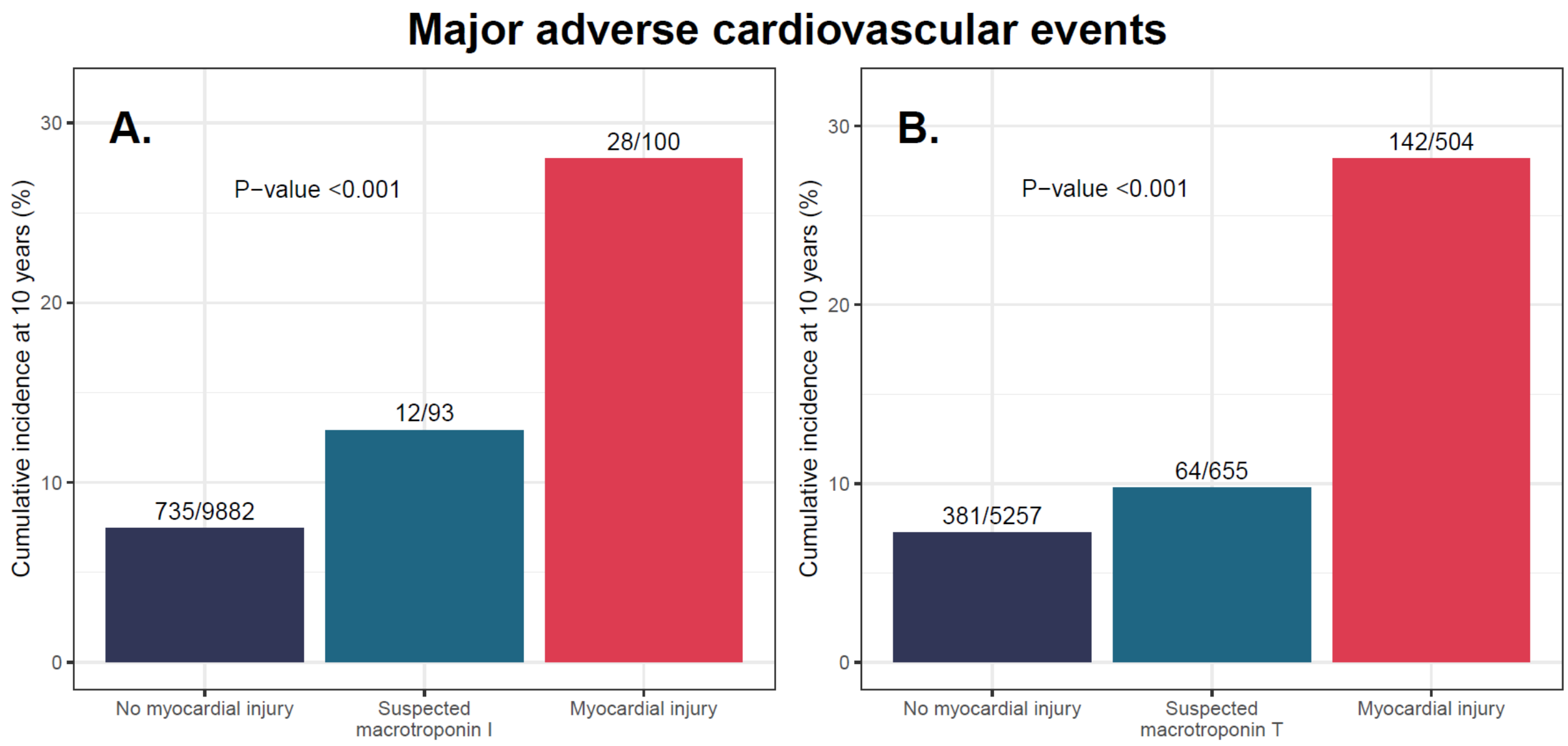

Supplemental Figure S10. Cumulative incidence of major adverse cardiovascular events at 10 years in participants with and without suspected macrotroponin I (A) and T (B) complexes in those with cardiac troponin concentrations above the limit of detection. The presence of a suspected macrotroponin was defined as a value above the sex-specific 99th percentile upper reference limit of cardiac troponin I or T where significant discordance (more than a three-fold difference) was observed between these assays.

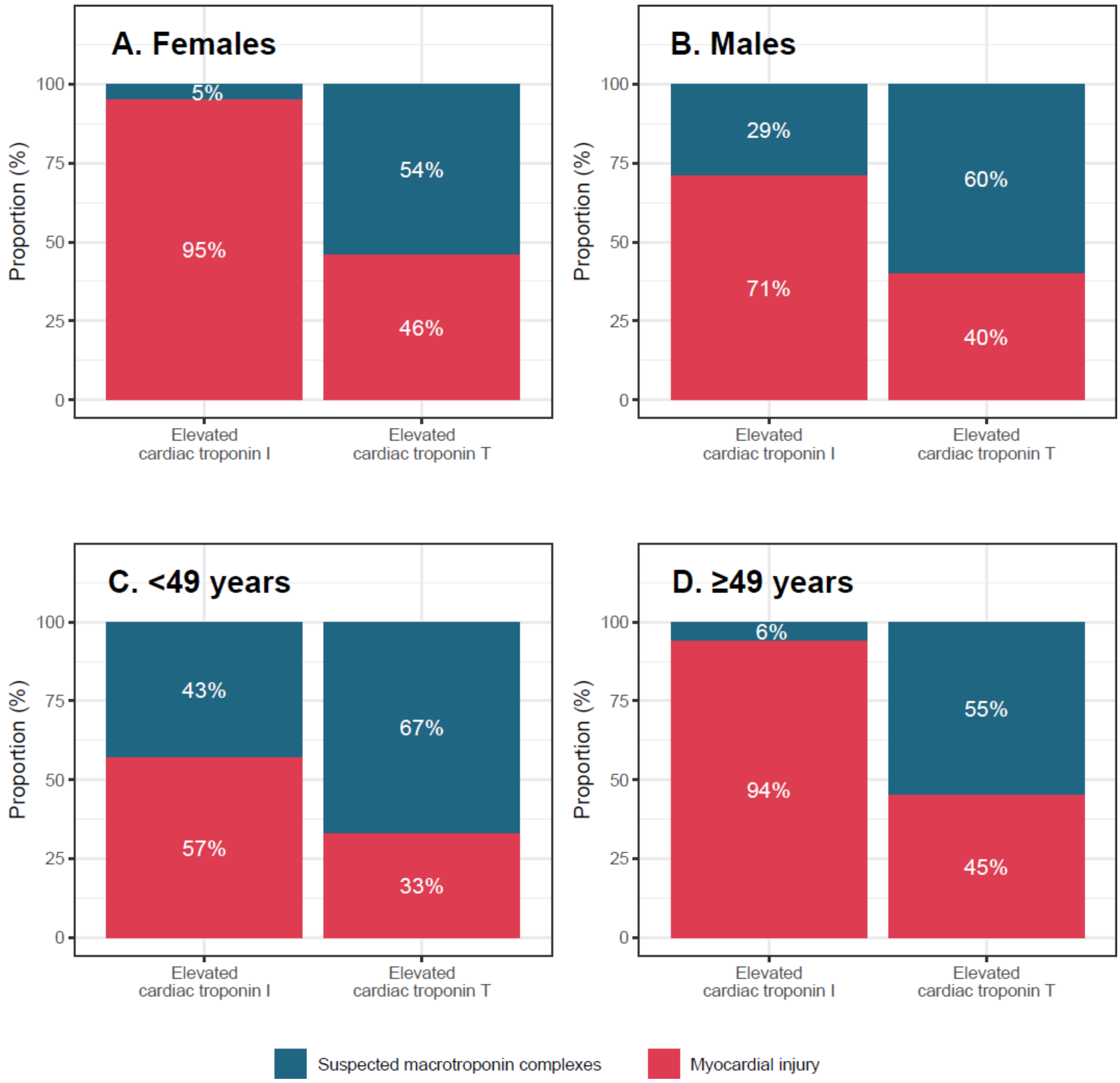

Supplemental Figure S11. Prevalence of suspected macrotroponin complexes stratified by sex and age in NHANES. The proportion of individuals with suspected macrotroponin complexes or myocardial injury among those with elevated cardiac troponin levels, stratified by sex (females [A] and males [B]) and by age (<49 years [C] and ≥49 years [D]). Suspected macrotroponin was defined as a value above the sex-specific 99th percentile upper reference limit of cardiac troponin I or T where significant discordance (more than a three-fold difference) was observed between these assays.

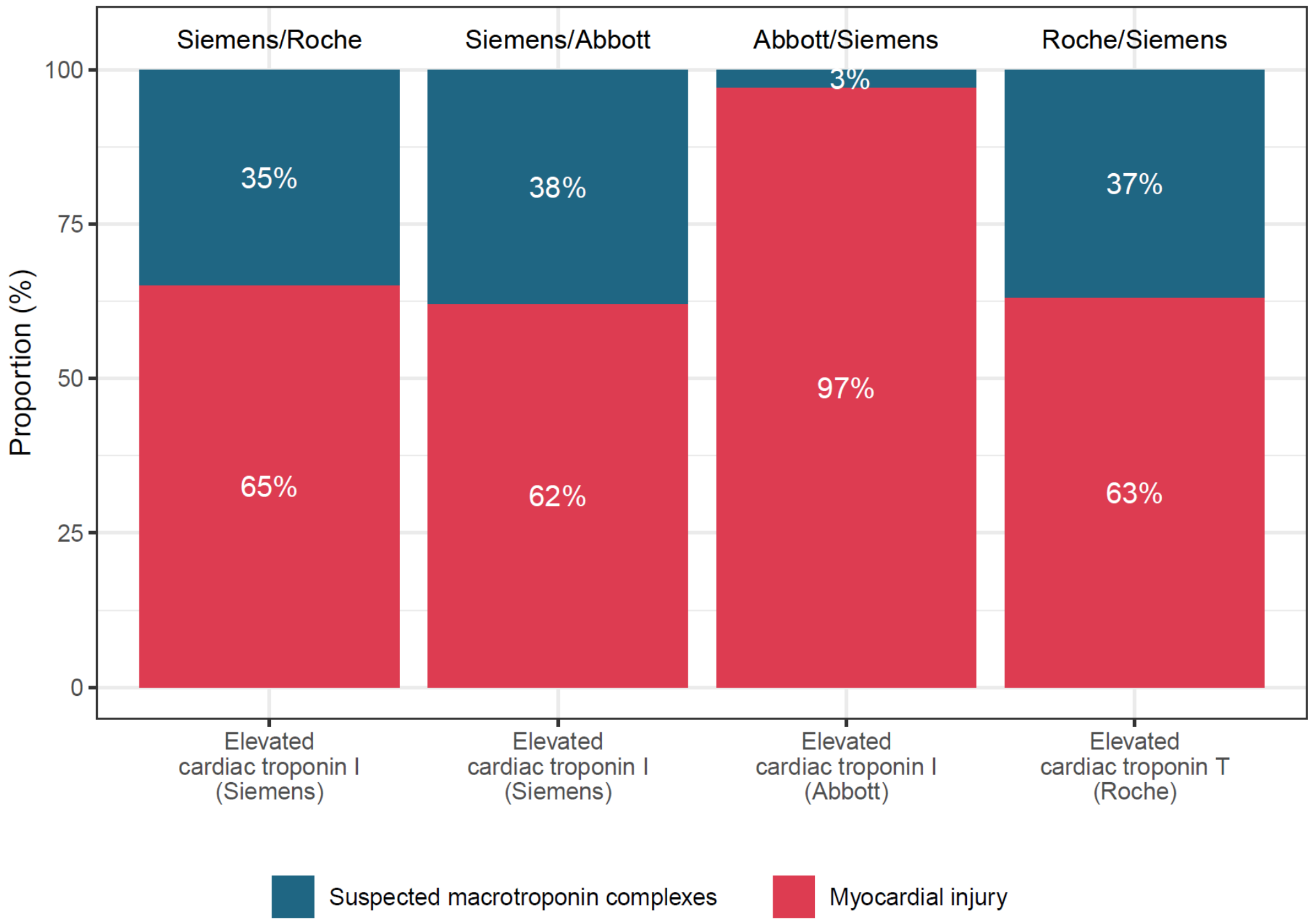

Supplemental Figure S12. Prevalence of suspected macrotroponin complexes in National Health and Nutrition Examination Survey based on various assay-to-assay comparisons. Suspected macrotroponin was defined as a value above the sex-specific 99th percentile upper reference limit of cardiac troponin I or T where significant discordance (more than a three-fold difference in absolute concentration) was observed between the assays. The prevalence estimate (number of US adults) of an elevated cardiac troponin I was 1% (2.4 million) using Abbott assay and was 1% (2.5 million) using the Siemens assay. The prevalence estimate of an elevated cardiac troponin T was 11% (21.6 million) using the Roche assay.

STROBE checklist

	Item No	Recommendation	Page No
**Title and abstract**	1	(*a*) Indicate the study's design with a commonly used term in the title or the abstract	2
		(*b*) Provide in the abstract an informative and balanced summary of what was done and what was found	2
**Introduction**			
Background/rationale	2	Explain the scientific background and rationale for the investigation being reported	3
Objectives	3	State specific objectives, including any prespecified hypotheses	3
**Methods**			
Study design	4	Present key elements of study design early in the paper	17 to 21
Setting	5	Describe the setting, locations, and relevant dates, including periods of recruitment, exposure, follow-up, and data collection	17 to 21
Participants	6	(*a*) *Cohort study*—Give the eligibility criteria, and the sources and methods of selection of participants. Describe methods of follow-up<br>*Case-control study*—Give the eligibility criteria, and the sources and methods of case ascertainment and control selection. Give the rationale for the choice of cases and controls<br>*Cross-sectional study*—Give the eligibility criteria, and the sources and methods of selection of participants	17 to 21<br>N/A<br>N/A
		(*b*) *Cohort study*—For matched studies, give matching criteria and number of exposed and unexposed<br>*Case-control study*—For matched studies, give matching criteria and the number of controls per case	N/A<br>N/A
Variables	7	Clearly define all outcomes, exposures, predictors, potential confounders, and effect modifiers. Give diagnostic criteria, if applicable	17 to 23
Data sources/ measurement	8	For each variable of interest, give sources of data and details of methods of assessment (measurement). Describe comparability of assessment methods if there is more than one group	17 to 21
Bias	9	Describe any efforts to address potential sources of bias	17 to 23
Study size	10	Explain how the study size was arrived at	Figure S1
Quantitative variables	11	Explain how quantitative variables were handled in the analyses. If applicable, describe which groupings were chosen and why	17 to 23
Statistical methods	12	(*a*) Describe all statistical methods, including those used to control for confounding	21 to 23
		(*b*) Describe any methods used to examine subgroups and interactions	21 to 23
		(*c*) Explain how missing data were addressed	23
		(*d*) *Cohort study*—If applicable, explain how loss to follow-up was addressed<br>*Case-control study*—If applicable, explain how matching of cases and controls was addressed<br>*Cross-sectional study*—If applicable, describe analytical methods taking account of sampling strategy	18 to 21<br>N/A<br>N/A
		(*e*) Describe any sensitivity analyses	21 to 23
**Results**			
Participants	13	(a) Report numbers of individuals at each stage of study—eg numbers potentially eligible, examined for eligibility, confirmed eligible, included in the study, completing follow-up, and analysed	Figure S1
		(b) Give reasons for non-participation at each stage	Figure S1
		(c) Consider use of a flow diagram	Figure S1
Descriptive data	14	(a) Give characteristics of study participants (eg demographic, clinical, social) and information on exposures and potential confounders	4, Table S1

		(b) Indicate number of participants with missing data for each variable of interest	Table S1
		(c) *Cohort study*—Summarise follow-up time (eg, average and total amount)	5 to 6
Outcome data	15	*Cohort study*—Report numbers of outcome events or summary measures over time	5 to 6
		Case-control study—Report numbers in each exposure category, or summary measures of exposure	N/A
		Cross-sectional study—Report numbers of outcome events or summary measures	N/A
Main results	16	(*a*) Give unadjusted estimates and, if applicable, confounder-adjusted estimates and their precision (eg, 95% confidence interval). Make clear which confounders were adjusted for and why they were included	5 to 8
		(*b*) Report category boundaries when continuous variables were categorized	N/A
		(*c*) If relevant, consider translating estimates of relative risk into absolute risk for a meaningful time period	N/A
Other analyses	17	Report other analyses done—eg analyses of subgroups and interactions, and sensitivity analyses	6 to 8
**Discussion**			
Key results	18	Summarise key results with reference to study objectives	9
Limitations	19	Discuss limitations of the study, taking into account sources of potential bias or imprecision. Discuss both direction and magnitude of any potential bias	13
Interpretation	20	Give a cautious overall interpretation of results considering objectives, limitations, multiplicity of analyses, results from similar studies, and other relevant evidence	9 to 14
Generalisability	21	Discuss the generalisability (external validity) of the study results	13
**Other information**			
Funding	22	Give the source of funding and the role of the funders for the present study and, if applicable, for the original study on which the present article is based	14
